# Toward functional integration of 3D‐printed meniscal scaffolds: A materials‐driven systematic review of preclinical evidence and translational framework

**DOI:** 10.1002/jeo2.70898

**Published:** 2026-09-07

**Authors:** Andrea Ferrero, Giuseppe Anzillotti, Pietro Conte, Simonetta Resta, Simone Micalizzi, Cecilia Cappelli, Nada Mansour, Paolo Oliva, Elizaveta Kon, Daniele D'Arrigo

**Affiliations:** ^1^ Department of Biomedical Sciences Humanitas University Pieve Emanuele Milan Italy; ^2^ IRCCS Humanitas Research Hospital Rozzano Milan Italy

**Keywords:** 3D printing, animal, biocompatible material, meniscus, surface property, tissue engineering

## Abstract

**Purpose:**

Meniscal injuries are among the most common knee disorders and a major risk factor for osteoarthritis. Although meniscectomy provides short‐term symptom relief, it irreversibly alters joint biomechanics, underscoring the need for regenerative strategies. Advances in additive manufacturing and biomaterials science have enabled three‐dimensional (3D)‐printed meniscal scaffolds with controlled architecture, tunable anisotropy and tailored biological functionality. This systematic review critically evaluates preclinical in vivo evidence on these strategies and identifies key factors influencing their functional integration and translational potential.

**Methods:**

A systematic literature search was conducted in PubMed, Web of Knowledge and Scopus up to September 2025, following Preferred Reporting Items for Systematic Reviews and Meta‐Analyses (PRISMA) guidelines. Included studies involved preclinical in vivo models of meniscectomy evaluating 3D‐printed meniscal scaffolds with reported mechanical and biological outcomes. Data extraction focused on biomaterials, fabrication techniques, scaffold design, mechanical performance and regenerative outcomes.

**Results:**

Twenty‐three studies met the inclusion criteria. Resorbable polymer‐based scaffolds, predominantly polycaprolactone, were most frequently investigated. Biofunctionalisation strategies—including cellularisation, biochemical cues and hydrogel–polymer composites—were widely employed. Most studies used rabbit models. Outcomes revealed a dynamic interplay between material composition, architecture, degradation kinetics and biological integration. An initial decline in mechanical properties was followed by progressive maturation, in some cases approaching native anisotropic behaviour and correlating with cellular infiltration and matrix deposition. Biofunctionalised scaffolds consistently demonstrated improved cartilage preservation compared with meniscectomy alone. However, heterogeneity in experimental design and reporting standards limited cross‐study comparability.

**Conclusion:**

3D‐printed meniscal scaffolds show promising preclinical potential for promoting tissue regeneration and preserving joint integrity. To bridge the gap between experimental findings and clinical application, this review introduces a structured reporting checklist identifying key material, structural and biological determinants of successful scaffold integration. By promoting methodological harmonization and standardized outcome assessment, this framework aims to enhance cross‐study comparability and support the stepwise clinical translation of next‐generation bioactive scaffolds for meniscal injuries.

**Level of Evidence:**

N/A.

## Graphical abstract

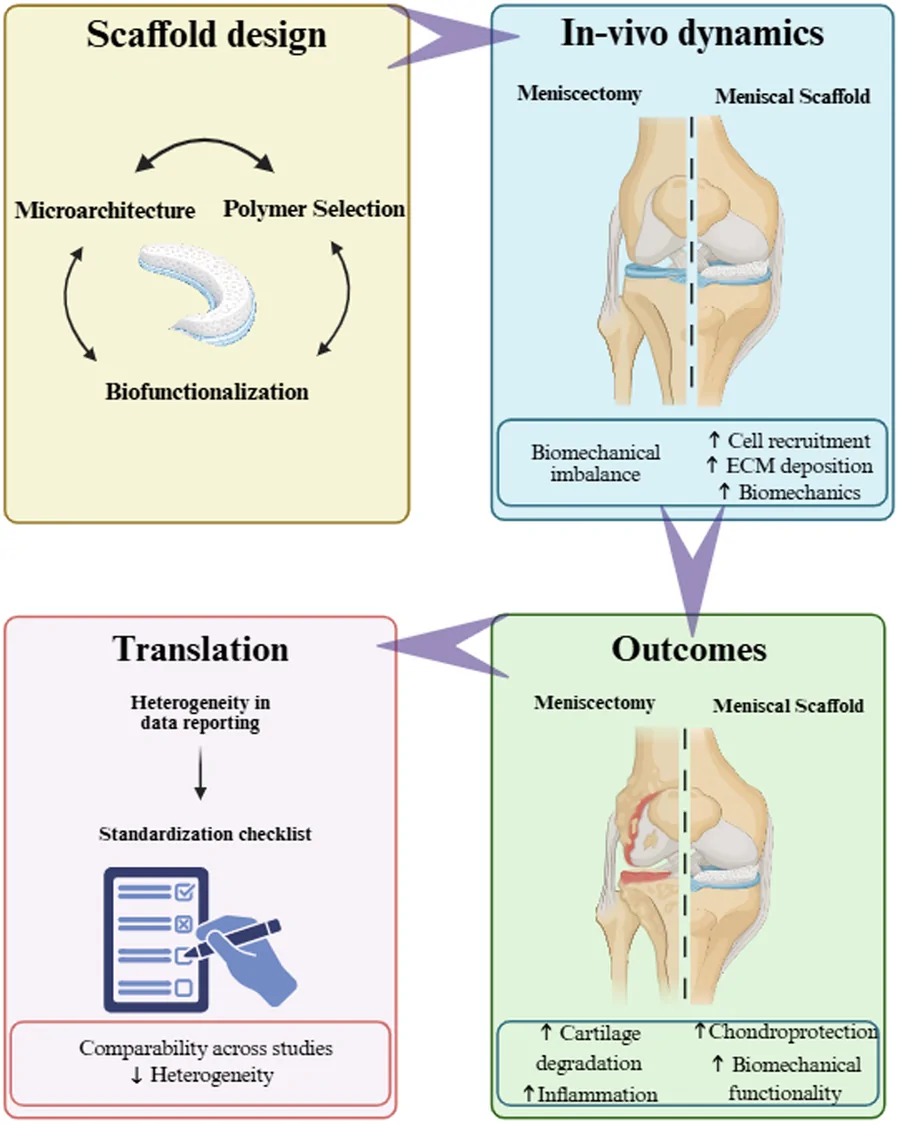

**Abbreviations**
3D: three‐dimensional
BM‐MSCs: bone marrow‐derived mesenchymal stem cells
CAD: computer‐aided design
COL I/COL II: Type I/Type II collagen
CT: computed tomography
CTGF: connective tissue growth factor
DA: Diels–Alder
DIW: direct ink writing
DLP: digital light processing
ECM: extracellular matrix
FDM: fusion deposition modelling
GAG: glycosaminoglycan
ICRS: International Cartilage Repair Society
IQR: interquartile range
KGN: kartogenin
MECM: meniscus extracellular matrix
micro‐CT: micro‐computed tomography
MRI: magnetic resonance imaging
MSCs: mesenchymal stem cells
NZW: New Zealand White (Rabbit)
OARSI: Osteoarthritis Research Society International
PCL: polycaprolactone (poly(ε‐caprolactone))
PCU: polycarbonate‐based polyurethane
PCU‐DA: polycarbonate‐based polyurethane with Diels–Alder crosslinking
PDGF‐BB: platelet‐derived growth factor‐BB
PLDLA: poly(L‐lactide‐co‐D,L‐lactide)
PLDLA‐TMC: PLDLA‐trimethylene carbonate copolymer
PLGA: poly(lactic‐co‐glycolic acid)
PNAGA: poly(N‐acryloyl glycinamide)
PNASC: poly(N‐acryloyl semicarbazide)
PRISMA: Preferred Reporting Items for Systematic Reviews and Meta‐Analyses
PS: polycaprolactone/silk fibroin
PU: polyurethane
PUA: polyurethane acrylate
PVA: polyvinyl alcohol
SD‐MSCs: synovium‐derived mesenchymal stromal cells
SYRCLE: SYstematic Review Center for Laboratory animal Experimentation
TGF‐β3: transforming growth factor beta 3
TMC: trimethylene carbonate

## INTRODUCTION

Meniscal tears are among the most common types of injury in the knee joint, resulting from both traumatic and degenerative processes, and are well‐recognized as risk factors for osteoarthritis progression because they are responsible for the biomechanical and biological destabilization of the knee joint. The limited self‐healing capacity of the meniscus, particularly in the inner avascular region, limits its spontaneous repair. In addition, the available clinical options are limited and cannot ensure complete functional recovery. For instance, meniscectomy, the gold standard treatment for years, provides short‐term symptom relief, but it irreversibly alters joint load transmission, increases contact stresses and impairs homoeostasis [49]. Therefore, regenerative strategies able to restore both structural integrity and biological function of the meniscus are necessary [40, 55]. In this context, biomaterial‐based scaffolds emerged not only as substitutes for the damaged tissues but as bioactive systems designed to modulate the biological environment and promote tissue regeneration. New‐generation biomaterials are in fact expected to coordinate cell recruitment, modulate inflammation and guide extracellular matrix (ECM) deposition to progressively transfer mechanical loads to the regenerating tissue [5, 23]. For load‐bearing tissues such as the meniscus, this requires integrating polymer chemistry, microarchitecture, degradation kinetics and mechanical properties. The scaffold's final performance therefore depends not only on bulk mechanical properties, but on the dynamic interplay between material composition, microstructure and host response.

Advances in material science and additive manufacturing technologies significantly expanded the possibilities to design effective meniscal scaffolds. Three‐dimensional (3D) printing allows the spatially controlled deposition of polymers, hydrogels and bioactive components, enabling the fabrication of constructs with tunable porosity, fibre orientation and anisotropic mechanical gradients [9, 18, 28, 53]. This is particularly relevant for the meniscus, whose native structure is characterized by circumferentially aligned collagen bundles that counteract hoop stresses and radial fibres preventing longitudinal splitting [2]. Therefore, 3D printing provides a platform to engineer scaffolds that can approximate the functional and biological anisotropy of native meniscal tissue. Clinically, 3D‐printed meniscal scaffolds would be primarily indicated for symptomatic post‐meniscectomy syndrome, for extensive or irreparable meniscal tears located in the avascular zone that are unsuitable for repair, and, more recently, for total meniscal replacement, representing an evolving alternative to meniscal allograft transplantation in appropriately selected patients [25]. These lesions are diagnosed clinically and confirmed by magnetic resonance imaging (MRI), which remains the reference standard for assessing meniscal tear pattern, location and extent prior to treatment planning.

Parallel to the 3D architecture, polymer selection represents another central determinant of scaffold behaviour. Resorbable aliphatic polyesters such as poly(ε‐caprolactone) (PCL) have been widely adopted due to their printability, biocompatibility and relatively slow hydrolytic degradation [8, 14]. However, their intrinsic hydrophobicity and limited bioactivity often require chemical or biological functionalization. Strategies including chemical modifications, cell seeding, incorporation of biochemical cues and composites with hydrogels have been explored to enhance cell–material interactions and guide cell differentiation [1, 22, 38, 42]. In parallel, elastomeric and non‐resorbable systems, such as polyurethane‐based networks and supramolecular hydrogels, have been investigated to provide prolonged mechanical support and improved compliance. In these systems, crosslinking density, segmental chemistry and molecular architecture critically influence stiffness, mechanical properties and degradation kinetics, thus shaping their biological response [16, 56].

Despite rapid technological progress, the clinical translation of meniscal scaffolds remains limited. First‐generation commercial resorbable and non‐resorbable implants demonstrated partial tissue ingrowth but inconsistent chondroprotective effects and mechanical performance, associated with notable failure rates in humans [13, 15, 29, 41]. A key challenge for these approaches lies in the coordinated balance between progressive biological integration and mechanical competence. Premature degradation may compromise load distribution before sufficient matrix deposition occurs, whereas excessive stiffness may induce stress shielding and impair physiological mechanotransduction. Understanding how material chemistry and scaffold architecture modulate these processes and their temporal evolution is therefore essential to design a rational and effective meniscal scaffold.

Although a number of reviews addressed meniscal tissue engineering and scaffold strategies, they provided broad overviews without systematically analysing the correlation between the biomaterial and the bioactivation strategies with the key parameters fundamental for the clinical translation of novel potential scaffolds, such as mechanical performance and chondroprotective efficacy [4, 15, 32, 57, 63]. Therefore, this systematic review aims to address these gaps by offering a focused, translation‐oriented analysis of 3D‐printed meniscal scaffolds. Specifically, we provide a detailed and quantitative evaluation of mechanical properties and chondroprotection‐related outcomes, including histological scoring and cartilage preservation. Beyond isolated metrics, we investigate the interplay between scaffold design parameters—such as polymer composition, functionalization strategies and architectural features—and their resulting mechanical and biological performance. In addition, we introduce a structured checklist to promote standardization in experimental design and reporting, facilitating cross‐study comparability. By integrating systematic data extraction with a material‐ and function‐centred perspective, this review aims to provide actionable insights to guide the development of next‐generation, load‐bearing 3D‐printed meniscal scaffolds for clinical application.

## MATERIAL AND METHODS

This systematic review adhered to the PRISMA (Preferred Reporting Items for Systematic Reviews and Meta‐Analyses) protocol guidelines [47]. A comprehensive literature search was performed in PubMed, Web of Knowledge and Scopus by two independent investigators. To cover the relevant literature, the following query was used for each database: ‘Menisc* AND (3D print* OR 3D OR bioprint*) AND (scaffold OR tissue engineering)’. The search included all studies published up to the beginning of September 2025. Reference lists of all included articles and relevant reviews were screened to identify further eligible studies. Manuscripts were selected according to predefined inclusion and exclusion criteria (Table 1), focusing on in vivo animal models and evaluating biomechanically characterized 3D‐printed meniscal scaffolds and reporting biological and/or functional outcomes. Reviews, meta‐analyses, expert opinions and conference abstracts/proceedings were excluded because these sources do not report original primary data and their inclusion could have resulted in double‐counting findings already captured through the primary preclinical studies identified by the search. Nevertheless, the reference lists of the most relevant narrative and systematic reviews were manually screened to identify any additional eligible primary studies that may not have been retrieved by the electronic search.

**Table 1:** Screening inclusion and exclusion criteria.

Inclusion criteria	Exclusion criteria
Preclinical in vivo studies using animal models of partial or total meniscectomy.	In vitro or in silico studies that do not involve animal experimentation.
Use of 3D printing or additive manufacturing techniques for scaffold fabrication.	Studies not involving 3D‐printed meniscal scaffolds.
Studies reporting mechanical testing of the implant (before and/or after implantation).	Absence of appropriate control or comparator groups.
Studies reporting biocompatibility, tissue integration, matrix deposition or regenerative evaluations.	Studies written in languages other than English.
	Reviews, meta‐analyses, expert opinions, conference abstracts or conference proceedings.

Abbreviation: 3D, three‐dimensional.

Titles and abstracts were independently screened by two reviewers in the first instance, followed by full‐text evaluation of potentially eligible studies. Discrepancies were resolved through discussion with a senior reviewer. Data were extracted independently by two authors using a standardized form, focusing on the following variables: model acquisition and design, biomaterial composition, 3D‐printing techniques, scaffold architecture, mechanical properties, animal models, control groups and biological response outcomes. The methodological quality of the included studies was independently evaluated by two reviewers using the SYRCLE Risk of Bias Assessment tool for animal studies [21]. This instrument assesses potential bias across domains, including random sequence generation, allocation concealment, blinding, incomplete outcome data, selective reporting and other potential sources of bias. Each domain was classified as: Yes, No or Unclear. Disagreements were resolved by consensus with a senior reviewer.

The extracted data were organized into structured summary tables to compare methodologies, results and findings across studies. When numerical data were presented only in graphical form, values were digitized using WebPlotDigitizer. Graphical representations were generated through the Matplotlib library in Python (Python Software Foundation).

The physiological reference ranges for mechanical properties were derived from values reported for native meniscus tissues in the five included studies that reported these parameters [6, 11, 33, 37, 59]. The values reported within these five primary studies were obtained under testing conditions directly comparable to those used for the corresponding scaffolds, whereas the wider literature summarized in reviews is affected by substantial heterogeneity in testing protocols [55]. Details of the data extraction procedure and compiled dataset are provided in Supporting Information S1: Table 1.

## RESULTS

**Figure 1:**
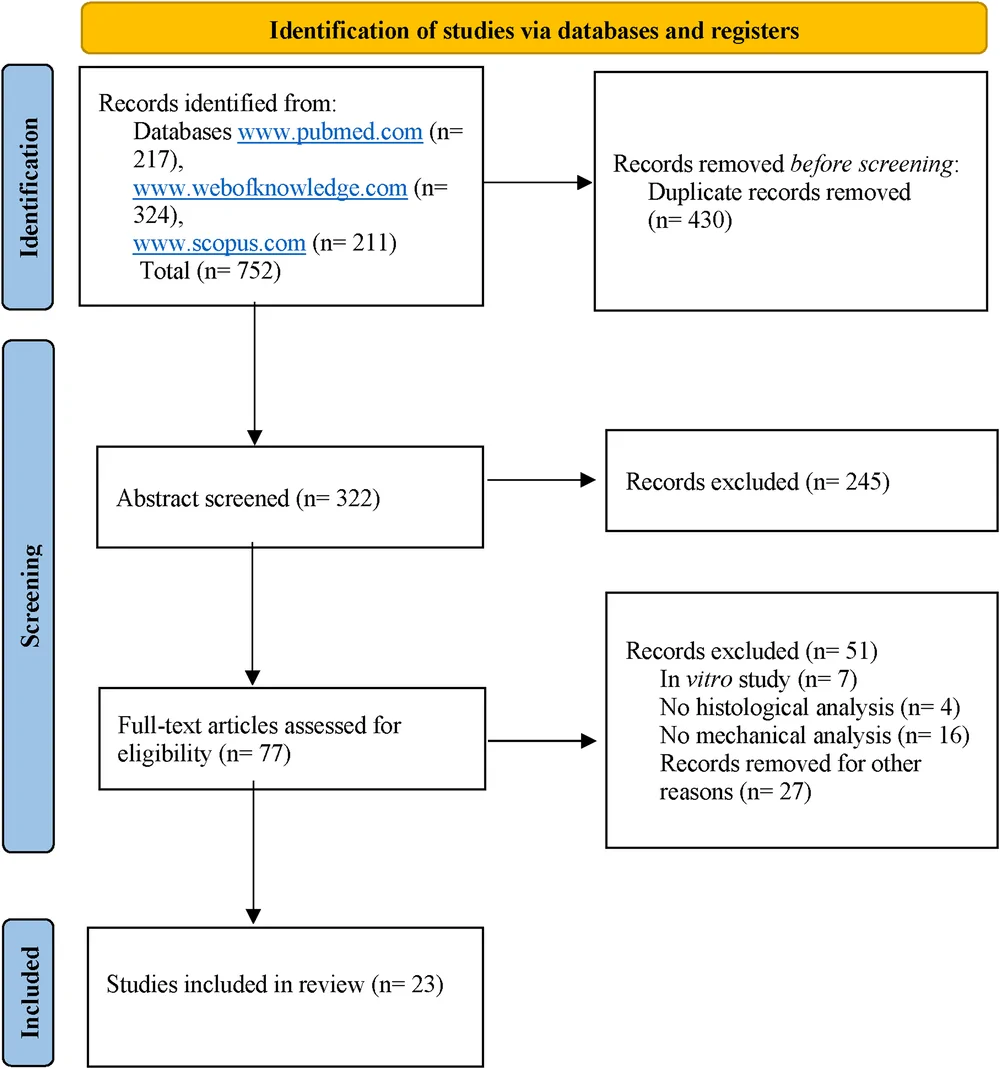
PRISMA flowchart summarizing the screening process for the systematic review. PRISMA, Preferred Reporting Items for Systematic Reviews and Meta‐Analyses.

The study selection process is summarized in Figure 1. A total of 752 articles were screened, of which 23 fulfilled the inclusion criteria and were included.

**Figure 2:**
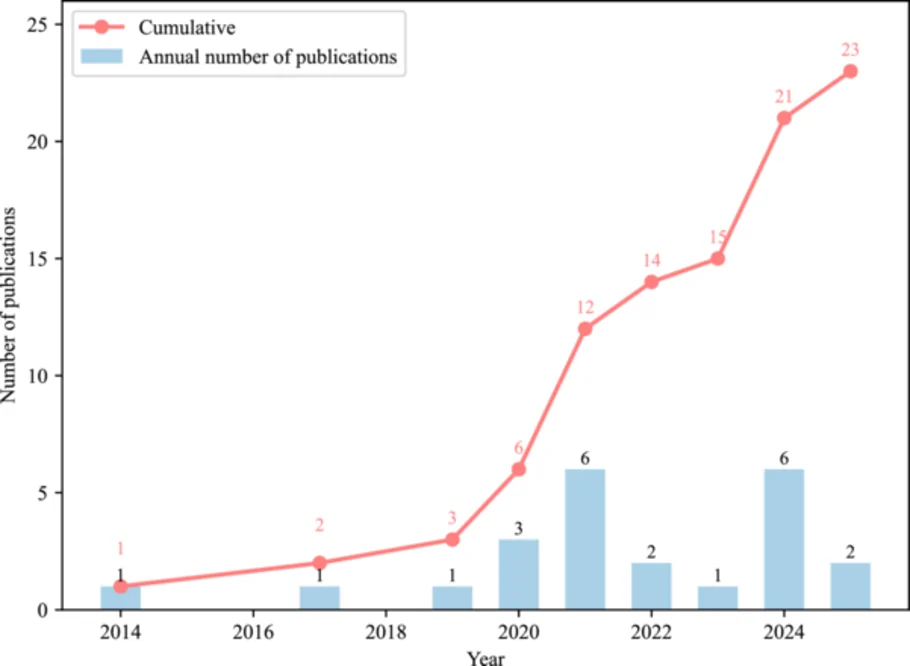
Publication trend from database inception until August 2025.

All the selected studies investigated key steps in the development of 3D‐printed meniscal scaffolds. Eleven out of 23 studies (48%) were published from 2022 onwards, indicating the rapid expansion of interest in bio‐fabricated meniscal substitutes (Figure 2). This growth reflects advances in biomaterial design and additive manufacturing technologies, enabling increasingly complex and bioactive scaffold architectures.

The main findings are summarized in Tables 2 and 3, where the included studies are categorized according to scaffold degradability into resorbable (17/23; 71%) [6, 12, 17, 20, 24, 27, 31, 33, 34, 35, 36, 37, 39, 58, 59, 67, 69] and non‐resorbable (6/23; 29%) [11, 61, 64, 65, 66, 68]. Animal models varied, and while small animal models predominated, rabbits were used in most of the studies (19/23; 82%) [6, 11, 12, 20, 24, 27, 33, 34, 35, 36, 37, 39, 59, 61, 64, 66, 67, 68, 69]. Large animal models were employed to better approximate joint loading and translational relevance and included sheep [31], goats [58] and dogs [65]. The scaffold performance was evaluated through mechanical testing, histological analysis, imaging and cartilage degeneration scores. However, reporting standards and quantitative metrics varied considerably, limiting direct comparison across studies.

**Table 2:** Main outcomes for resorbable scaffolds.

Study	Scaffold type	3D printing technique	Animal model	Treatments tested	Main scaffold findings (↑ = increase, ↓ = decrease, ↔ = comparable, *significant difference)	
First author, year, ref.	Polymer; Bioactivation		Number, species, weight, age	Experimental groups and controls	Mechanical properties	Other outcomes
Zhang ZZ et al. 2017 [69]	PCL seeded with BM‐MSCs	FDM	72 NZW rabbits, 3 [kg]	*Exp. Group*: PCL + BM‐MSC *Controls*: PCL (not seeded), Meniscectomy, Sham	Pre‐implant: ￪ tensile modulus versus control ￪compressive aggregate modulus versus control Post‐implant: *At 3 and 6 months* PCL + BM‐MSC ￪ tensile modulus versus controls* ￪ compressive aggregate modulus versus cell‐free scaffolds*	Tissue regeneration: ￪ tissue integration, no sign of tear or gap formation PCL + BM‐MSC versus non‐seeded *At 6 months* ￪ Col I/II* in PCL + BM‐MSCs versus non‐seeded ￪ proteoglycans in PCL + BM‐MSCs versus non‐seeded Cartilage preservation: ↓ ICRS scores in PCL + BM‐MSCs versus non‐seeded and meniscectomy* ↓ Mankin scores in PCL + BM‐MSCs versus non‐seeded and meniscectomy* ↔ lymphocyte infiltration within new tissue and foreign body reaction around the polymer scaffolds versus non‐seeded Zonal differentiation: *At 6 months* ↔ cell phenotypes within implants at different zones PCL + BM‐MSCs versus native meniscus Cartilage preservation: ↓ cartilage degeneration femur and tibia PCL + BM‐MSCs versus non‐seeded and meniscectomy Vascularization: ￪ in the outer regions of the new meniscal tissue ↔ new vessel ingrowth PCL + BM‐MSCs versus native ↔ integration with surrounding tissues PCL + BM‐MSCs versus native
Sun Y et al. 2020 [58]	PCL hydrogel seeded with BM‐MSCs	OPUS system	12 adult sheep 6 months old	*Exp. Group*: PCL + BM‐MSCs + μS of CTGF or TGFβ3 *Controls*: PCL + BM‐MSCs	Post‐implant: *At 3 months* ￪ tensile modulus versus controls ￪ aggregate modulus versus controls ￪ ultimate tensile strength versus controls ￪ radial tensile versus controls Regional variations (outer versus inner) ￪ bidirectional tensile testing* *At 6 months* ￪ tensile and compressive modulus with respect to 3 months	Tissue regeneration: ￪ Col 1–rich fibrous tissue in the outer region versus control ￪ Col 2–rich cartilaginous tissue in the inner region versus control ￪ GAG versus control Zonal differentiation: Region‐specific matrix resembling the native meniscus Cartilage preservation: ￬ Mankin versus control ￬ ICRS versus control Vascularization: Not specified
Sun Y et al. 2021 [59]	PCL seeded with SD‐MSCs	OPUS system	12 rabbits 3 months old	*Exp. Group*: PCL + SD‐MSCs hydrogel + μS of CTGF and TGFβ3 + Mg^2+^ *Control*: PCL + SD‐MSCs hydrogel	Post‐implant: *At 3 months* ￪ tensile modulus (within physiological range) versus control ￪ aggregate modulus versus control ￪ ultimate tensile strength ￪ radial tensile properties	Tissue regeneration: ￪ Col 1/3 deposition versus control ￪ GAG production versus control Zonal differentiation: Region‐specific matrix resembling the native meniscus Cartilage preservation: ￬ Mankin scores versus control ￬ ICRS scores versus control Vascularization: ￪ peripheral blood vessel ingrowth versus controls (↔ to native)
Du M et al. 2024 [12]	PCL seeded with BM‐MSCs	FDM	48 NZW rabbits 3–6 months old	*Exp. Group*: (GSDP‐TEM) + BM‐MSCs *Controls*: USDP, USSP, Native	Pre‐implant: ￪ GSDP tensile modulus in the circumferential direction like native ￬ GSDP tensile modulus lowest in the radial direction like native compressive modulus >40 MPa. USSP intermediate values USDP lowest mechanical performance.	Tissue regeneration: *At 3 and 6 months* ↔ Cell type and distribution versus native meniscus ↔ ECM composition distribution versus native meniscus ￪ SERPIN E1, BMP‐6 and BMPR genes (chondrogenesis) Zonal differentiation: ↔ meniscus regeneration with heterogeneous cell differentiation Cartilage preservation: ￬ Mankin score versus scaffold controls ￬ ICRS versus scaffold controls Vascularization: Not specified
Chen M et al. 2019 [6]	PCL seeded with fibro‐chondrocytes	FDM	100 NZW rabbits (2.5–3 [kg]) 3–6 months old	*Exp. Group*: PCL‐hydrogel‐MECM + Fibrochondrocyte hybrid scaffold *Controls*: Meniscectomy, PCL scaffold, PCL hydrogel, Sham surgery	Pre‐implant: compressive moduli out of physiological range Post‐implant: *At 3 and 6 months* ￪ tensile moduli versus PLC + hydrogel and versus PLC alone ￪ compressive moduli versus PLC + hydrogel and versus PLC alone ￪ tensile moduli PLC + hydrogel versus PLC alone ￪ compressive moduli versus PLC alone ↔ tensile modulus versus sham	Tissue regeneration: *At 3–6 months* ↔ tissue appearance of neo‐menisci versus native with fibroblast‐like and chondrocyte‐like cells ￪ Col I in the outer region versus unseeded ￪ Col II in the outer region versus unseeded ↔ Collagen content versus sham group ￬ GAG content un‐seeded Zonal differentiation: ￪ fibroblast‐like and chondrocyte‐like cells in the inner region versus un‐seeded Vascularization: Not specified
Jeong HJ et al. 2021 [24]	PCL seeded with fibro‐chondrocytes	FDM	NZW rabbits (3 [kg]) 1.5–3 months old	*Exp. Group*: PCL + composite powder (PCL, NaCl) + Fibro‐chondrocytes *Control*: PCL	Pre‐implant: ￪ tensile modulus in control versus experimental group Post‐implant: *At 1.5 and 3 months* 1.5 month: ￬ respect to preimplant in tensile and compressive modulus 3 months: ￪ tensile modulus and compressive modulus versus control	Tissue regeneration: ↔ elongated fibroblast‐like cells in both groups ↔ round chondrocyte‐like cells in both groups ￪ Col I in both groups ￪ Coll II in both groups Zonal differentiation: ↔ cellular shape and size to native meniscus Vascularization: Not specified
Guo W et al. 2021 [17]	PCL seeded with MECM	FDM	40 NZW rabbits (3.0 [kg]) 20 Sheep 3–6 months old	*Exp. Group*: PCL‐MECM (large pore) *Controls*: Meniscectomy, autograft, sham	Pre‐implant: ￪ tensile modulus small pore aligned scaffolds (within the physiological range) versus controls ￬ compressive modulus of large‐pore scaffolds versus controls (in physiological range) Better anisotropy in large‐pore aligned scaffold versus small pore.	Tissue regeneration: ￪ Col‐1–rich fibrous regions in the outer zone versus controls ￪ Col‐2–rich cartilaginous regions in the inner zone versus controls Zonal differentiation: Meniscus‐like heterogeneous staining and cell distribution in neo‐menisci Cartilage preservation: ↔ Mankin score versus sham ￬ Pauli score Vascularization: Not specified
Li H et al. 2021 [33]	PCL seeded with MECM	FDM	12 NZW rabbits (8 months old, 2.5–3.0 [kg]) 3 months old	*Exp. Group*: PCL + MECM + μS KGN *Controls*: Meniscectomy, PCL, PCL + MECM	Pre‐implant: All scaffolds showed compression ↔ or ￪ modulus values versus native tissue. ￪ tensile modulus PCL + MEMC + KNG µS versus controls ￬ tensile modulus respect to native for all groups	Tissue regeneration: ￪ Col 1–rich fibrous tissue in the outer region ￪ GAG Zonal differentiation: ↔ tissue organization versus native meniscus Cartilage preservation: ￬ Ishida score Vascularization: Not specified
Lee CH et al. 2014 [31]	PCL+ Growth Factors	FDM	11 sheep, 2‐ to 5‐years‐old, 3 months old	*Exp. Group*: PCL + PLGA µS of CTGF and TGFβ *Controls*: Meniscectomy, PCL	Post‐implant: *At 3 months* ↔complex modulus versus native meniscus ↔ viscoelastic properties versus native meniscus ↔ tensile Young's modulus versus native meniscus ↔ friction coefficient versus native meniscus. ￪ pull‐out strength versus native partial grafts	Tissue regeneration: Homogeneous regeneration of tissue versus control Zonal differentiation: ↔ native‐like fibrocartilage with a zone‐specific tissue phenotype: a collagen‐rich, aligned fibrous matrix in the outer zone; a fibrocartilaginous matrix in the intermediate zone; and a cartilaginous matrix in the inner zone. Cartilage preservation: ↔ articular cartilage alteration in femoral or tibial condyle versus native Vascularization: Not specified
Hao L et al. 2022 [20]	PCL+ Growth Factors	Dual Nozzle Bio‐printing	32 rabbits, 2.5–3 [kg], 2 knees per rabbit used; 3–6 months old	*Exp. Group*: PCL + PDGF‐BB and KGN µS	Pre‐implant: ￬ compressive modulus versus controls (in physiological range) ￬ tensile modulus versus controls	Tissue regeneration: ￪ GAG and collagen, ↔ microscopic resemblance to native tissue ￪ cellular integration versus control Zonal differentiation: ￪ uniform shape versus control Cartilage preservation: ￬ OARSI versus meniscectomy and PCL + PDGF‐BB Vascularization: Not specified
Li Y et al. 2020 [36]	PCL; Chemical Functionalization	Wet‐Electro‐spun	*Exp. Group*: PCL + silk fibroin + Sr2+ *Control*: Sham	*Exp. Group*: PCL + silk fibroin + Sr2+ *Controls*: Sham	Pre‐implant: ↑ tensile modulus versus P and SP ↑ compressive modulus versus P and SP	Tissue regeneration: *At 6 months* ↑ meniscus‐like tissue formation in SP‐Sr group ↑ ordered collagen fibres arrangement ↑ Col I/II distribution Zonal differentiation: ↑ differentiation in SP‐Sr group (fibroblast‐like outer cells, limited chondrocyte‐like cells in inner region) Cartilage preservation: ↓ cartilage degeneration in SP‐Sr group versus meniscectomy Vascularization: ↓ blood vessel distribution in neo meniscus
Li Z et al. 2020 [37]	PCL; Chemical Functionalization	FDM	18 NZW rabbits (2.7–3.2 [kg]); 3–6 months old	*Exp. Group*: PS‐L7 *Controls*: Meniscectomy, PS	Pre‐implant: ￪ tensile moduli in PS‐L7 versus PS ￪ compressive moduli in PS‐L7 versus PS Post‐implant: *At 3 months* ￪ tensile moduli* versus controls (in physiological range) ￪ compressive moduli* versus controls	Tissue regeneration: ￪ tissue regeneration versus control Zonal differentiation: Not specified Cartilage preservation: ￬ cartilage degeneration versus PS, ￬ Inflammatory markers after 1 week over time in all groups. Vascularization: Not specified
Ma H et al. 2024 [39] 3	PCL; Chemical Functionalization	FDM	Rabbits 3–6 months	*Exp. Group*: PCL‐Fib *Controls*: Meniscectomy, PCL	Pre‐implant: ￪￪ tensile modulus versus controls (in physiological range) ￪￪ compressive modulus versus controls	Tissue regeneration: *At 3 months* ￪ tissue regeneration versus PCL ￪ collagen fibres versus PCL Zonal differentiation: Not specified Cartilage preservation: ￪ smoother neo‐meniscus surface versus PCL ￪ Pauli score (*) versus PCL ￬ Mankin score versus meniscectomy group ￪ time for joint space narrowing in PCL group Vascularization: Not specified
Li J et al. 2024 [35]	PCL; Chemical Functionalization	Extrusion	36 rabbits (3.5 ± 0.5 [kg]) 3–6 months, right knee	*Exp. Group*: PG‐Pg (PCL +graphene) + (PVA/glycerol) *Controls*: Meniscectomy, sham	Pre‐implant: ￬ tensile and compressive modulus versus native Post‐implant: *After 3 and 6 months* ￪ aggregate moduli versus pre‐implant ￪ tensile moduli versus pre‐implant ￪ ultimate tensile load versus pre‐implant ￬ permeability versus pre‐implant	Tissue regeneration: *At 3 and 6 months* ↔ Col I versus native ↔ Col II versus native Zonal differentiation: ￪ chondrocyte disorganization in the PG‐Pg group versus native tissue Cartilage preservation/degeneration: ￬ ICRS PG‐Pg versus meniscectomy* ￬ Mankin PG‐Pg versus meniscectomy* ￪ chondroprotection PG‐Pg versus meniscectomy ￬ inflammatory indices in all groups (gradually over time) ￬ MMP1, MMP13, TIMP1 in PG‐Pg versus meniscectomy. Vascularization: Not specified
Zhang Q et al. 2022 [67]	PCL; Hydrogel	FDM	39 rabbits (2.5–3.0 [kg])	*Exp. Group*: PCL + PNAGA *Controls*: Meniscectomy, PCL, sham	Pre‐implant: ￬ compressive modulus versus control ￬ tensile and compressive modulus versus native	Tissue regeneration: ↑ scaffold integrity and position retention versus PCL and meniscectomy No scaffold fracture (improved mechanical stability versus PNAGA‐only scaffold). Zonal differentiation: Not specified Cartilage preservation: ↑ chondroprotection versus meniscectomy and PCL ↓ OARSI score versus meniscectomy and PCL ↑ ICRS score versus meniscectomy and PCL ↔ cartilage surface appearance to sham Vascularization: Not specified
Li H et al. 2025 [34]	PCL; Hydrogel	FDM	30 rabbits (2.5–3 [kg])	*Exp. Group*: PCL + hydrogel + Mg^2+^ (PSM‐Mg) *Controls*: Meniscectomy, PCL + hydrogel (PSM), sham	Post‐implant: *At 3 and 6 months* ￪ tensile modulus PSM versus PCL + hydrogel ￪ compressive modulus	Tissue regeneration: *At 3 months* ￪ proliferation of neo‐meniscal tissue versus meniscectomy and PSM Zonal differentiation: Cell phenotype region‐specific distribution in experimental group ↔ ECM region‐specific deposition versus native meniscus Cartilage preservation/degeneration: ￬ cartilage damage versus meniscectomy and PSM ￬ WORMS in PSM‐Mg versus PSM but ￪ versus sham ￬ OARSI in PSM‐Mg versus PSM and meniscectomy ￬ Mankin in PSM‐Mg versus PSM and meniscectomy ￬ ICRS in PSM‐Mg versus PSM and meniscectomy Vascularization: Not specified
Komatsu D et al. 2024 [27]	PLDLA‐TMC; Cellularization	FDM	9 Rabbits	*Exp. Group*: pl‐ PLDLA‐TMC + Cells *Controls*: Meniscectomy, PLDLA‐TMC	Pre‐implant: ￪ tensile values versus controls (in physiological range) Post‐implant: *At 3 months* ￬ tensile moduli (reaching 1/8 of the original value by 3 months)	Tissue regeneration: ↑ meniscus‐like tissue formation in both scaffold groups ↑↑ mature fibrocartilage regeneration with MSC‐loaded scaffold, approaching negative control; no polymer remnants detected (vs. polymer fragments in scaffold‐only group) Zonal differentiation: ↑ restored zonal architecture (surface/middle/deep zones) in PLDLA‐TMC + Cells‐scaffold versus control Cartilage preservation: ↑↑ chondroprotection with MSC‐loaded scaffold, AC morphology almost identical to negative control; preserved tidemark and subchondral bone architecture Vascularization: Not specified

Abbreviations: 3D, three‐dimensional; BM‐MSCs, bone marrow‐derived mesenchymal stem cells; COL‐1/COL‐2, Collagen Type I/Collagen Type II; CTGF, connective tissue growth factor; DA, Diels–Alder; FDM, fusion deposition modelling; GSDP‐TEM, gradient‐sized diamond‐pored microstructure tissue‐engineered meniscus; KGN, kartogenin; MECM, meniscus extracellular matrix; MSCs, mesenchymal stem cells; NZW Rabbit, New Zealand White Rabbit; PCL, polycaprolactone; PCL‐Fib, polycaprolactone fibrosin; PCU, polycarbonate‐based polyurethane; PDGF‐BB, platelet derived growth factor BB; PLDLA, poly(L‐lactide‐co‐D,L‐lactide); PNAGA, poly(N‐acryloyl glycinamide); PS, polycaprolactone/silk fibroin; PVA, polyvinyl Alcohol; TMC, trimethylene carbonate; USDP, uniform diamond structured; USSP, uniform square structured; µS, microsphere.

**Table 3:** Main outcomes for non‐resorbable scaffolds.

Study	Scaffold type	3D printing technique	Animal model	Treatments tested	Main scaffold findings (↑ = increase, ↓ = decrease, ↔ = comparable, *significant difference)	
First author, year, ref.	Polymer; Bioactivation		Number, species, weight, age	Experimental groups and controls	Mechanical properties	Outcomes
Ding G et al. 2022 [11]	PU; KGN	DLP	New Zealand White Rabbit (2.7–3.5 [kg])	*Exp. Group*: PU/PDA + KGN (PU‐5 K‐50) *Controls*: Meniscectomy, PU	Pre‐implant: ↔ tensile and compressive modulus of PU/PDA/KNG versus native (in physiological range) Post‐implant: *At 24 weeks* ↔ tensile and compressive modulus of PU/PDA/KNG versus native (in physiological range)	*At 3 months* Tissue regeneration: Formation of typical fusiform morphology of cells in experimental group Zonal differentiation: Not specified Cartilage preservation: ↓ ICRS in PU/PDA + KGN versus PU* Vascularization: Not specified
Yan W et al. 2024 [65]	PUA; Hydrogels + drugs	DIW	Beagle canines 3 months	*Exp. Group*: PUA elastomeric skeleton + WPUA hydrogel matrix + drugs (diclofenac sodium and lipid‐soluble KGN) *Controls*: Meniscectomy, PUA elastomeric skeleton, Native control group	Pre‐implant: ￪ tensile modulus versus control	*At 3 months* Tissue regeneration: ￪ cellular proliferation versus PUA ↔ fibrocartilage regeneration, fibro‐chondrocytes and GAG deposition to native control Cartilage preservation: ↓ OARSI versus control ↓ GAG deposition ↔ cartilage erosion versus native Zonal differentiation: ↑ COL I in lateral region and ↑ COL II in the medial and intermediate regions in PUA/WPUA/drugs versus control Vascularization: Not specified
Tian F et al. 2024 [61]	PUA; Hydrogel and drugs	DIW	18 adult rabbits (male, 3 [kg]) 2 months	*Exp. Group*: PUA elastomeric skeleton + WPUA hydrogel matrix + drugs (melatonin) *Controls*: Meniscectomy	↓ tensile and compressive moduli compared to other PUA scaffold, due to nanoindentation data extraction	*At 2 months* Tissue regeneration: Not specified Cartilage preservation: Mild cartilage erosion in MFC and MTP with superficial cartilage fibrillation. ↓ OARSI scores versus meniscectomy group* Zonal differentiation: Not specified Vascularization: Not specified
Xu Z et al. 2023 [64]	PNASC/PNAGA	Extrusion	18 rabbits (2.5–3.0 [kg]) 1–3 months	*Exp. Group*: GMP‐ PNASC/PNAGA *Controls*: Meniscectomy, Sham	Pre‐implant: Tensile modulus in physiological range Low compressive modulus ￪ Young's modulus in peripheral region (within physiological range)	*At 4 and 12 weeks* Tissue regeneration: Not specified Cartilage preservation: Cartilage surface intact, little abrasion in GMP‐PNASC/PNAGA versus sham ↓ OARSI scores versus meniscectomy* Zonal differentiation: Not specified Vascularization: Not specified
Zhang Q et al. 2024 [68]	PNASC/PNAGA	DLP	42 rabbits (male, 2.5–3.0 [kg]) 1.5–3 months old	*Exp. Group*: pl‐ PNASC/PNAGA group *Controls*: Meniscectomy, pl‐PNASC group, Sham	Pre‐implant: Pre‐stretching significantly varies tensile strength and modulus, when aligned with fibre orientation, bringing values into the physiological range. *At 28 days* Mechanical properties remained stable in PBS (good durability)	*At 12 weeks* Tissue regeneration: Not specified Cartilage degeneration: ↓ OARSI score versus pl‐PNASC and meniscectomy ￪ OARSI versus sham group ↔ FCs and TPs in the pl PNASC/PNAGA group ↔ superficial matrix and normal cartilage morphology as sham group Zonal differentiation: Not specified Vascularization: Not specified
Yang X et al. 2025 [66]	PCU; DA bonds	FDM	22 rabbits (male, 2.5–3.0 [kg])	*Exp. Group*: pl‐ PCU‐DA *Controls*: Meniscectomy, Sham	Pre‐implant: Compressive modulus and tensile modulus out of physiological values	*At 4 and 12 weeks* Scaffold maintains structural integrity and appropriate positioning Tissue regeneration: Not specified Cartilage preservation: Cartilage surface intact, smooth and continuous in PCUDA‐1 group versus meniscectomy ↔ OARSI scores to sham OARSI scores ↓ versus meniscectomy Zonal differentiation: Not specified Vascularization: Not specified

Abbreviations: COL‐1/COL‐2, Collagen Type I/Collagen Type II; DA, Diels–Alder; DIW, direct ink writing; DLP, digital light processing; FDM, fusion deposition modelling; KGN, kartogenin; NZW Rabbit, New Zealand White Rabbit; PCU, polycarbonate‐based polyurethane; PNAGA, poly(N‐acryloyl glycinamide); PNASC, poly(N‐acryloylsemicarbazide); PUA, polyurethane acrylate.

### Scaffold design and production process

**Figure 3:**
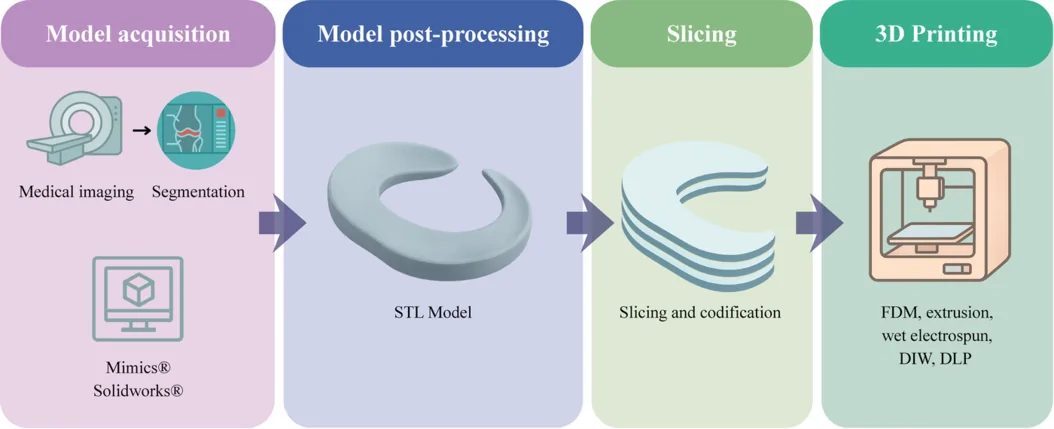
3D models of the meniscal tissue are generated through two main approaches: reconstruction from medical imaging and 3D model development with computer‐aided design (CAD) software. They are then sliced and converted into printing instructions for the selected fabrication platform. Figure created using Canva (Canva Pty Ltd.). 3D, three‐dimensional; DIW, direct ink writing; DLP, digital light processing; FDM, fusion deposition modelling; STL, stereolithography.

The production phases necessary to obtain 3D‐printed scaffolds, including model acquisition and image processing, printing and scaffold functionalization, were systematically analysed (Figure 3).

#### 3D scaffold model acquisition and processing

The acquisition and digitization of the 3D digital model to be 3D‐printed is the first crucial phase. The architecture (fibre orientation, mesh and pore size) of this digital model, in fact, plays a primary role in determining the scaffold's final properties, influencing the cell phenotype, adherence, migration, proliferation and differentiation [56]. For instance, smaller pore sizes were demonstrated to induce chondrogenic differentiation, facilitating cartilaginous matrix formation [45].

Scaffold geometries were derived either from medical imaging datasets or computer‐aided design (CAD) models. Imaging‐based approaches included micro‐computed tomography (micro‐CT) in six studies [6, 17, 34, 61, 66, 67], MRI in three [35, 39, 69], CT scan in one [24] and laser scanning in one [31]. These datasets were manually segmented to reconstruct meniscal anatomy; however, technical details of imaging resolution and post‐processing were generally not reported. Notably, native meniscal tissue has low inherent radiographic contrast and is not directly visualized by standard micro‐CT/CT without dedicated soft‐tissue staining or contrast‐enhancement protocols [46]. Since the six micro‐CT/CT‐based studies did not provide detailed information on these preprocessing steps, the methods used for meniscal boundary delineation remain difficult to assess from the published reports. On the other hand, CAD‐based designs, used in the remaining studies [20, 24, 27, 31, 34, 37, 58, 59, 61, 64, 69], generated wedge‐shaped or anatomically inspired constructs with circumferential and radial fibre organization to mimic the native tissue anisotropy.

Hybrid strategies were also described. Sun Y et al. [58] combined medical images from unspecified source to design the external meniscal geometry with CAD‐generated internal architecture. Du M et al. [12] developed gradient pore designs integrating diamond and square microstructures to achieve spatial variation in internal architecture.

#### Polymer selection and biofunctionalization

**Figure 4:**
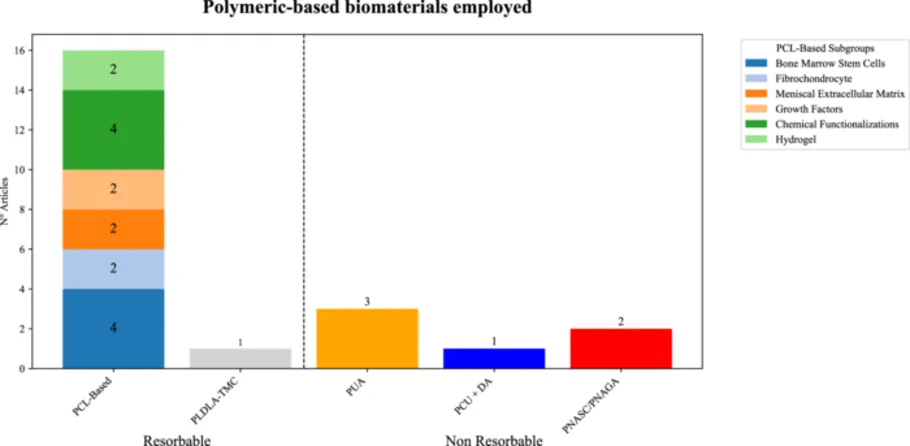
Polymer‐based materials used for creating resorbable and non‐resorbable scaffolds, and the number of studies in which they were used. For PCL scaffolds, different colours indicate the specific biofunctionalization strategies adopted. BMSCs, bone marrow stem cells; DA, dopamine; PCL, polycaprolactone; PLDLA‐TMC, poly(L‐lactide‐co‐D,L‐lactide)‐trimethylene carbonate; PNAGA, poly(N‐acryloyl glycinamide); PNASC, poly(N‐acryloylsemicarbazide); PCU, polycarbonate urethane; PUA, polyurethane acrylate.

Polymers were classified as either *resorbable*, intended to gradually degrade and be replaced by native tissue, or *non‐resorbable*, designed to provide long‐term structural support (Figure 4). Most scaffolds incorporated additional biofunctionalization strategies to enhance biomechanical properties.

##### Resorbable scaffolds

Poly(ε‐caprolactone) (PCL), predominantly used for resorbable scaffolds [6, 12, 17, 20, 24, 31, 33, 34, 35, 36, 37, 39, 58, 59, 67, 69], is a polymer widely used as a structural biomaterial, characterized by a melting point of approximately 60°C, high biocompatibility, a long biodegradation time and versatility in terms of tailoring molecular weight, shape and mesh organization [57]. The only alternative resorbable system employed the PLDLA‐TMC (poly(L‐co‐D,L‐lactic acid‐co‐trimethylene carbonate) terpolymer), which allows modulation of mechanical properties, crystallinity and degradation kinetics [27].

Given the intrinsic hydrophobicity of PCL, multiple studies implemented strategies to enhance cell adhesion and regenerative response. Cellularization was the most common approach (6/16 studies), using heterologous bone marrow‐derived mesenchymal stem cells (BM‐MSCs) [12, 58, 69], synovium‐derived mesenchymal stromal cells (SD‐MSCs) [59] or fibrochondrocytes [6, 24]. Cells were expanded for up to three passages prior to seeding on the scaffold, but seeding density was reported only in one study [12]. Spatially controlled delivery of biological cues to reproduce the meniscal zonal architecture was achieved by encapsulating BM‐MSCs and growth factors within hydrogel phases integrated into the PCL scaffold [58]. A refinement of this scaffold in a second study included reduced fibre spacing and incorporation of magnesium ions, thereby enhancing angiogenesis and peripheral vascular ingrowth [59].

Acellular biofunctionalization strategies aimed to improve host cell recruitment and matrix deposition. Meniscal extracellular matrix (MECM) was integrated within the PCL backbone [17, 37] or combined with poly(lactic‐co‐glycolic acid) (PLGA) microspheres loaded with kartogenin (KGN) to promote fibrochondrocyte differentiation [33]. Growth factors, such as PDGF‐BB [20], connective tissue growth factor (CTGF) and transforming growth factor beta 3 (TGF‐β3) [31], were integrated either within bioinks to be co‐printed with PCL [20] or by tethering microspheres onto the scaffold surface [31]. Hydrogel–PCL composites and supramolecular networks were also employed to improve mechanical performance and cell differentiation [34, 67].

Additional chemical modifications included silk fibroin crosslinking [36, 37], fibrinogen coating [39] and graphene‐containing composites to increase cell adhesion, spreading and proliferation [35].

##### Non‐resorbable scaffolds

Non‐resorbable scaffolds were based on polyurethane acrylate (PUA), poly(N‐acryloyl semicarbazide) (PNASC)/Poly(N‐acryloyl glycinamide) (PNAGA) hydrogels and polycarbonate‐based polyurethane (PCU‐DA).

PUA‐based scaffolds which feature high flexibility and chemical resistance were frequently combined with bioactive hydrogels to deliver melatonin [61], KGN [11, 65] or diclofenac [65] to promote regeneration and modulate inflammation. Surface functionalization with polydopamine improved biocompatibility [11], while dynamic oxime‐urethane bonds conferred ‘self‐healing’ properties [61]. PNASC/PNAGA hydrogels [64, 68], characterized by strong hydrogen bonding and high‐water retention, were processed into composite scaffolds with enhanced hydration and mechanical strength. Finally, PCU‐DA scaffolds exploited Diels–Alder (DA) crosslinking to achieve a temperature‐responsive network that supports printing precision while maintaining physiological stability [66].

#### 3D‐printing techniques

**Figure 5:**
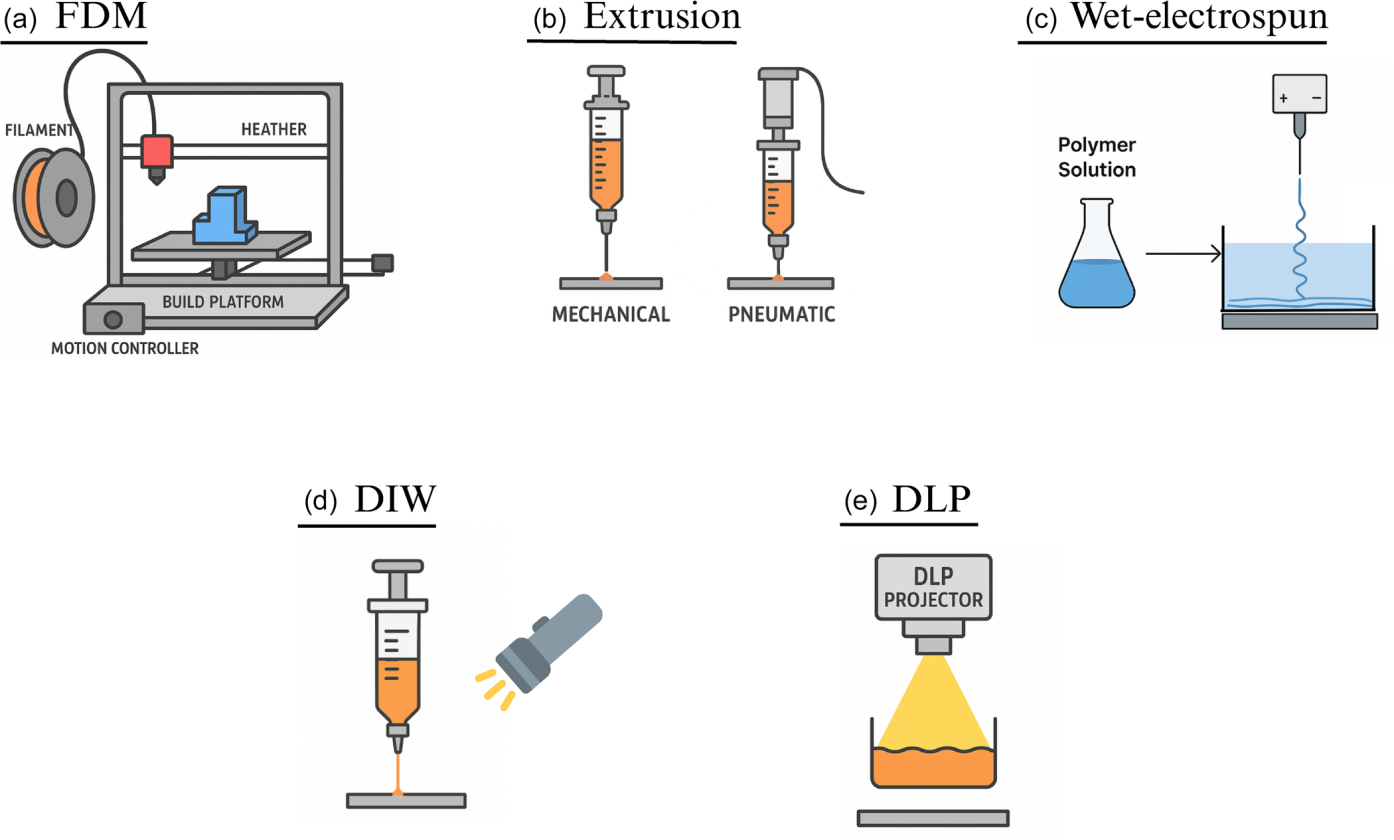
Schematic representation of the main additive manufacturing techniques employed for scaffold fabrication: (a) FDM, based on the extrusion of melted thermoplastic filaments (≈ 150–400 μm in diameter); (b) Extrusion‐based printing, performed through mechanical or pneumatic dispensing; (c) Wet‐electrospinning, enabling the deposition of polymer solutions under an electric field; (d) DIW involving extrusion of viscoelastic inks with subsequent curing; and (e) DLP based on photopolymerization of resin layers using a projected light source. Figure created using Canva (Canva Pty Ltd.). DIW, direct ink writing; DLP, digital light processing; FDM, fusion deposition modelling.

Five additive manufacturing approaches were employed across the included studies (Figure 5): fused deposition modelling (FDM), extrusion‐based bioprinting, wet‐electrospun, direct ink writing (DIW) and digital light processing (DLP). The selection of the technique was primarily based on the polymer properties and the need to preserve bioactivity.

FDM was the most frequently used method and was applied to print PCL [6, 12, 17, 24, 31, 33, 34, 37, 39, 67, 69], PCU‐DA [66] and PLDLA‐TMC scaffolds [27]. Reported operational parameters varied across the studies, with printing rate ranging from 1.3 to 7 mm/s, extrusion pressure between ≈550 and 800 kPa and nozzle temperatures from 85°C to 180°C, depending on the polymer. Multi‐material extrusion allowed the integration of hydrogel phases both unloaded and enriched with growth factors, allowing the spatially controlled delivery of bioactive cues [20, 58, 59].

DIW was used to fabricate elastic PUA scaffolds through the extrusion of gel‐like materials at room temperature followed by post‐curing, enabling precise control of microarchitecture [61, 65]. Composite scaffolds based on PCL–graphene [35] and PNASC/PNAGA [64] were developed using extrusion‐based systems with subsequent curing or drying steps to restore mechanical integrity. Two studies by Ding et al. [11] and Zhang Q et al. [68] employed DLP, a high‐resolution technology based on UV curing of resins, to produce PU/PDA + KGN and PNASC/PNAGA scaffolds. Through wet electrospinning, micrometre‐scale scaffolds made of PCL, silk fibroin and strontium ions were produced, resulting in randomly oriented fibre networks supporting cell proliferation [36].

### Mechanical performance over time and scaffold characterization

Mechanical characterization was mainly performed through uniaxial or multiaxial tensile and compressive tests. Additional parameters, such as permeability, friction coefficients, porosity and dimensional stability, were not reported systematically in all the studies.

**Figure 6:**
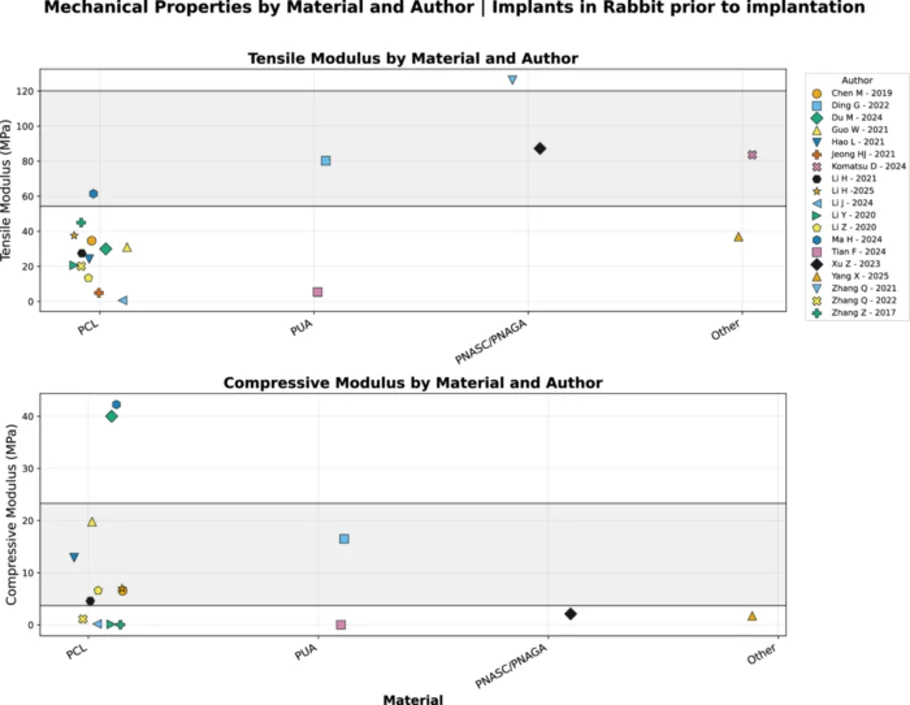
Mechanical properties are grouped by material and author, and each coloured symbol represents data from a single study. The upper panel shows the tensile modulus (MPa), while the lower panel shows the compressive modulus (MPa). The grey bands indicate the reference range expressed as IQR calculated from the literature for the tensile (54.3–120.1 MPa) and for the compressive modulus (3.7–23.3 MPa) (Supporting Information S1: Table 1). The compressive value reported by Li J et al. [35] corresponds to the aggregate modulus. The category ‘other’ includes PLDLA‐TMC and PCU. IQR, interquartile range; KGN, kartogenin; PCU, polycarbonate‐based polyurethane; PLDLA‐TMC, poly(L‐lactide‐co‐D,L‐lactide)‐trimethylene carbonate; PU/PDA, polyurethane/polydopamine; PUA, polyurethane acrylate.

Mechanical analyses were variably performed prior to implantation [6, 11, 12, 17, 20, 24, 27, 33, 34, 35, 36, 37, 39, 61, 64, 66, 67, 69] or both pre‐ and post‐implantation [6, 11, 24, 27, 35, 37, 59, 69]. The most reported parameters were tensile modulus, compressive modulus and aggregate modulus. For cross‐study comparability, Figure 6 presents the pre‐implantation tensile and compressive properties, while Supporting Information S1: Table 2 reports all the mechanical values extracted from the literature—including those not derived from rabbit samples.

PCL‐based scaffolds generally exhibited tensile moduli between 15 and 40 MPa, with one stiffer construct exceeding 60 MPa and approaching the physiological range [39] (reported to vary from 54.3 to 120.1 MPa). The other resorbable material, PLDLA‐TMC, showed higher tensile values compared to PCL. Greater values, closer to the physiological range, were observed in non‐resorbable scaffolds composed of PUA or PNASC/PNAGA, which also demonstrated anisotropic behaviour [11, 33]. This indicated a higher ability to reproduce native tissue–level stiffness than resorbable scaffolds. Tensile modulus values within the physiological range were reported in four studies [11, 27, 39, 64], while compressive modulus was reported in seven [6, 11, 17, 20, 33, 34, 37]. Simultaneous in‐range tensile and compressive moduli were achieved only by the PU/PDA + KGN scaffold [11] (Figure 6).

**Figure 7:**
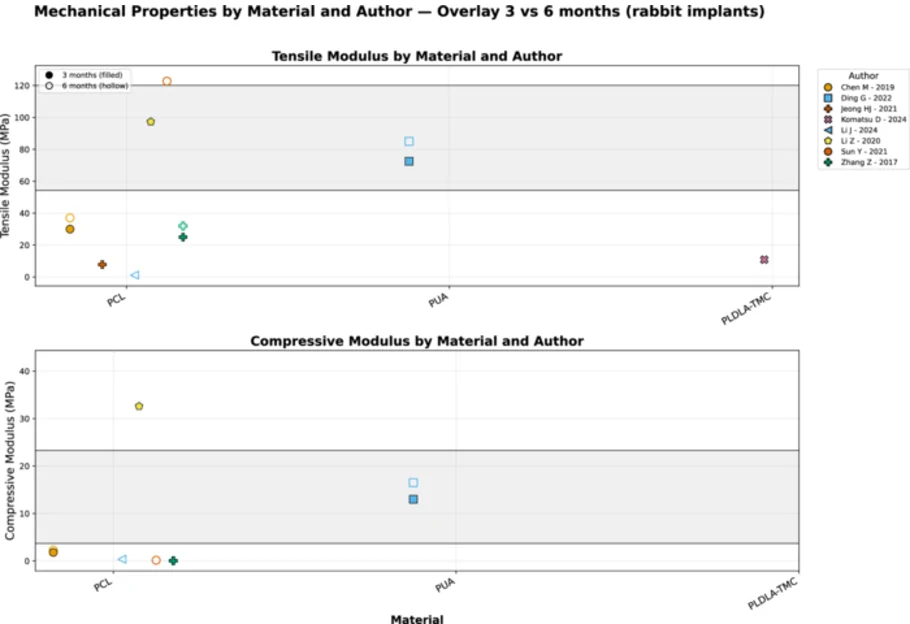
Mechanical properties of scaffolds at 3 (filled symbols) and 6 (hollow symbols) months after implantation, grouped by material and author, and each coloured symbol represents data from a single study. The upper panel shows the tensile modulus (MPa), while the lower panel shows the compressive modulus (MPa). The grey bands indicate the reference range expressed as IQR calculated from the literature for the tensile (54.3–120.1 MPa) and for the compressive modulus (3.7–23.3 MPa) (Supporting Information S1: Table 1). The compressive values reported by Li J et al. and Sun Y et al. correspond to aggregate modulus [35, 59]. The category ‘other’ includes PLDLA‐TMC. IQR, interquartile range; PLDLA‐TMC, poly(L‐lactide‐co‐D,L‐lactide)‐trimethylene carbonate.

Figure 7 summarizes the mechanical properties at 3 (filled symbols) and 6 (hollow symbols) months after implantation. Across studies, both PCL‐ and PUA‐based scaffolds exhibited lower mechanical properties initially, shortly after implantation, followed by a successive increase over time. This trend is consistent with early structural weakening associated with integration and, in PCL scaffolds, degradation processes. The subsequent compensation, and in some cases enhancement, of these values was likely due to effective tissue integration and, in PCL scaffolds, to cellular infiltration and ECM deposition [6, 11, 69]. Among the evaluated constructs, only the PU/PDA + KGN scaffold achieved in‐range tensile and compressive moduli simultaneously at follow‐up [11].

### In vivo evaluations

In vivo evaluation was central to assessing the capacity of 3D‐printed meniscal scaffolds to support tissue regeneration and preserve articular cartilage. All the studies investigated these aspects, reporting no major scaffold‐related failures. Two perioperative deaths were described in one study and were attributed to anaesthesia or surgical procedures rather than scaffold‐related factors [31].

#### Implantation and fixation strategies

Most scaffolds were implanted in the medial knee compartment, with surgical procedures reported in 78% (18/23) of the studies and minor variations due to anatomical differences among animal models. Total medial meniscectomy was performed in most of the cases, while two studies adopted partial meniscectomy, preserving 5%–10% of the native meniscus to provide an anchoring site for scaffold fixation [31, 33]. Access to the joint was most commonly achieved (15/18) by a medial parapatellar approach [11, 12, 17, 20, 24, 27, 34, 36, 39, 58, 64, 66, 67, 68, 69], while less invasive parameniscal exposures were occasionally used to reduce soft tissue disruption [11, 35]. In one case, a medial femoral condylectomy was performed to facilitate scaffold positioning and fixation, a more invasive but mechanically robust technique [31]. Scaffolds were sutured to the anterior and posterior horn insertions and to the joint capsule. In some cases, transtibial tunnels enhanced stabilization; [33, 35, 37] fixation details were not specified in three studies [33, 65, 68]. Postoperatively, animals were allowed free mobilization, simulating early weight‐bearing conditions that may influence scaffold stability and integration. None of the included studies reported fixation‐related complications such as scaffold extrusion, suture failure or loss of fixation, even if postoperative fixation integrity was not always explicitly assessed or reported as a discrete outcome.

#### Macroscopic and histological evaluation of the scaffold after in vivo implantation

All studies assessed tissue morphology by haematoxylin–eosin and ECM composition by Toluidine Blue (59%) and/or Safranin O (47%). Immunohistochemistry for collagen types I and II provided insight into zonal matrix organization and meniscus‐like tissue stratification [11, 12, 20, 24, 34, 37, 58, 65, 69].

Scaffold implantation generally promoted fibroblast and chondrocyte proliferation, glycosaminoglycan (GAG) deposition and region‐specific matrix organization. Resorbable scaffolds enabled progressive integration and guided tissue regeneration, whereas non‐resorbable constructs primarily provided mechanical support and chondroprotection, with more limited matrix remodelling.

Unmodified PCL scaffolds showed limited cell infiltration and modest matrix deposition [34, 67, 69]. In contrast, BM‐MSC–seeded constructs consistently enhanced fibrocartilage formation and zonal organization, characterized by fusiform fibroblast‐like cells in the outer region and rounded fibrochondrocyte‐like cells in the inner region [12, 58, 59, 69]. The detection of Type I collagen (COL I) and newly formed blood vessels in the outer layer and Type II collagen (COL II) with cartilaginous lacunae in the inner layer further supported the formation of a native‐like meniscal architecture [6, 24, 59].

Microstructural design emerged as a key determinant of cell fate: scaffolds with gradient diamond‐shaped pore architectures promoted spatially controlled differentiation and region‐specific ECM deposition, more closely reproducing native meniscal organization [12].

Biochemical functionalization further modulated regenerative outcomes. Growth factors such as CTGF and TGF‐β3 promoted zonal fibrocartilage formation [31], while PDGF‐BB enhanced endogenous cell recruitment and stratified tissue organization [20]. Scaffolds incorporating meniscus‐derived ECM [17, 33] supported zonal cellular organization and increased cell density in a sheep model [33]. Hybrid constructs combining PCL with hydrogel phases and Mg‐doped bioactive glass nanospheres sustained region‐specific fibrochondrogenesis and type II collagen deposition [34].

Protein‐based coatings (fibrin/fibrinogen) were associated with improved collagen organization and reduced inflammatory response compared with unmodified PCL [37, 39]. Other functionalization strategies showed mixed outcomes: graphene functionalization supported collagen I/II expression but without major architectural improvement [35], whereas PCL/silk fibroin/Sr^2+^ scaffolds showed poor cellular organization, likely reflecting suboptimal mechanical properties [36].

PLDLA scaffolds seeded with BM‐MSCs showed mature fibrocartilage and native‐like architecture, whereas cell‐free scaffolds exhibited thinner matrix and predominantly fibroblastic tissue [27].

Non‐resorbable PUA‐based hydrogels displayed abundant fibrochondrocytes, intense GAG staining and robust fibrocartilage formation, thanks to KGN‐mediated chondrogenic signalling [11, 65].

To summarize these outcomes, biofunctionalization consistently enhanced tissue ingrowth, zonal organization and collagen deposition across all the biomaterials employed. Neovascularization in the outer region was reported in several studies, indicating progressive integration with the host tissues. Only one study described a mild foreign‐body reaction to unmodified PCL, which diminished as degradation progressed over time [69].

#### Articular cartilage preservation

**Figure 8:**
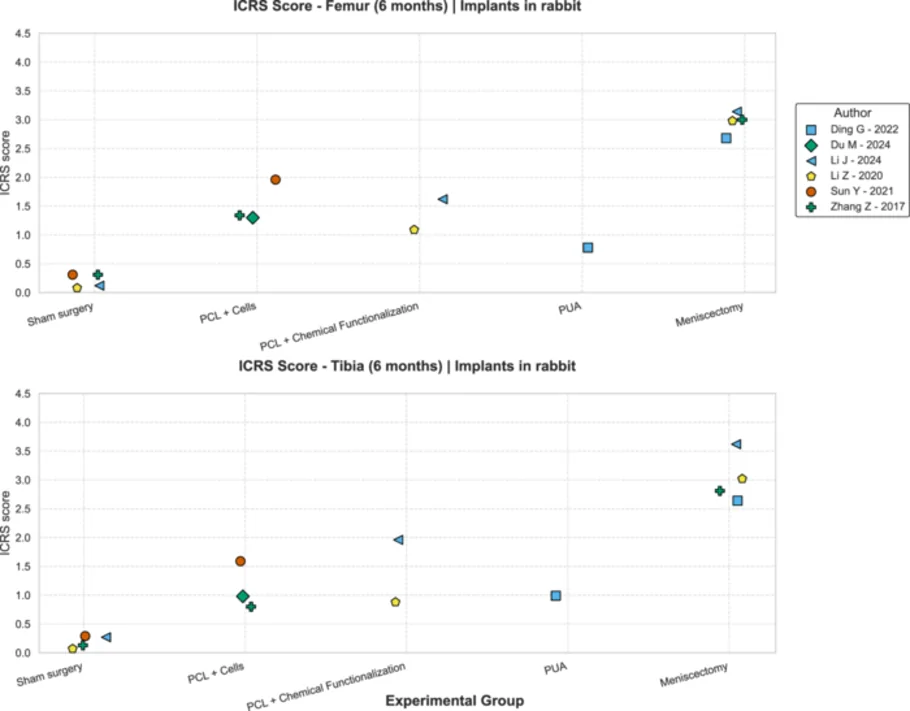
International Cartilage Repair Society (ICRS) scores at 6 months post‐implantation evaluated in the femur (upper) and tibia (lower), grouped by experimental condition. Control groups: sham surgery and meniscectomy. Each combination of symbol and colour represents a different study. PCL, polycaprolactone (poly(ε‐caprolactone); PUA, polyurethane acrylate.

**Figure 9:**
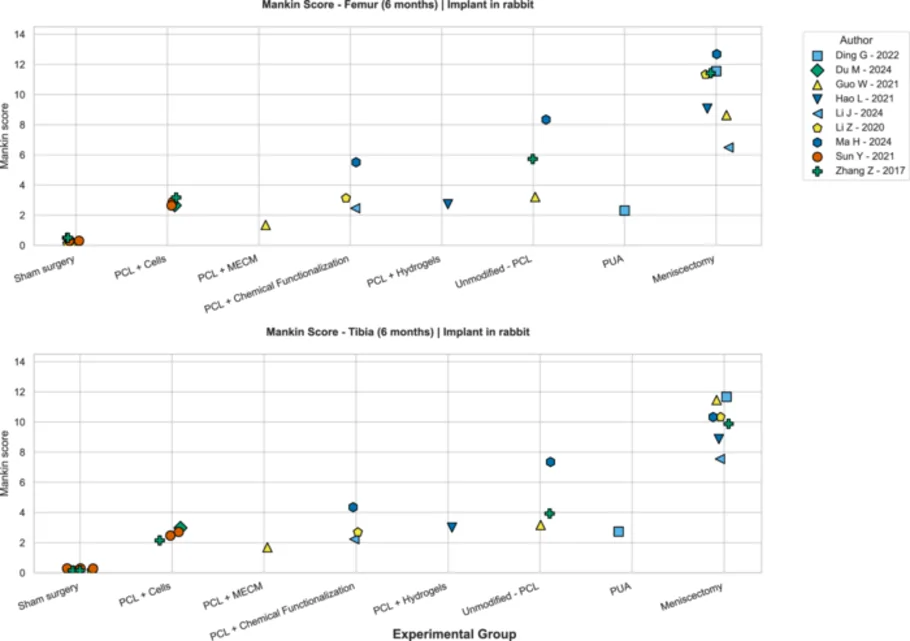
Mankin scores at 6 months post‐implantation evaluated in the femur (upper) and tibia (lower), grouped by experimental condition. Control groups: sham surgery and meniscectomy. Each combination of symbol and colour represents a different study. MECM, PCL, polycaprolactone (poly(ε‐caprolactone); PUA, polyurethane acrylate.

**Figure 10:**
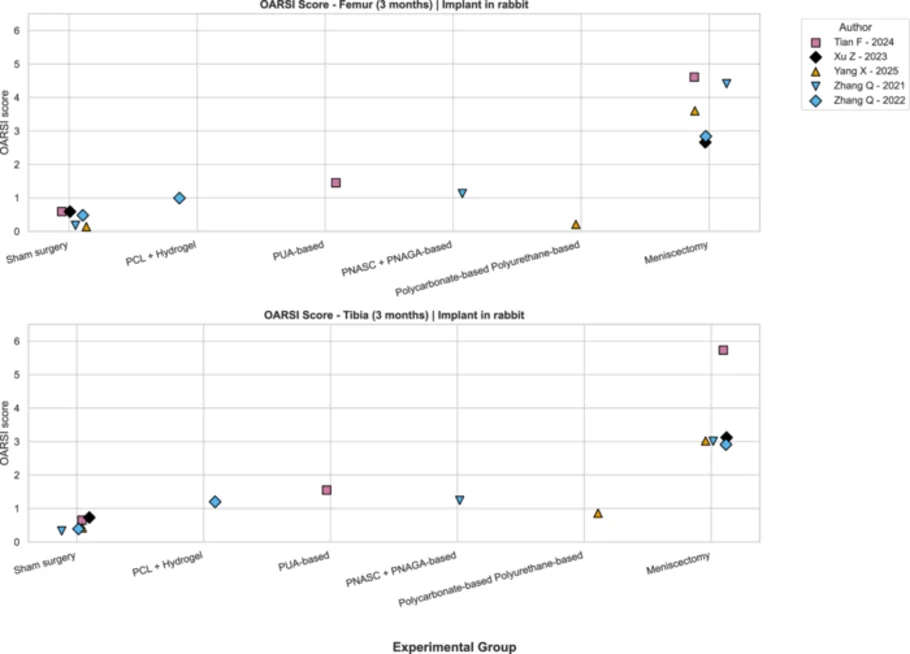
Osteoarthritis Research Society International (OARSI) scores at 6‐month follow‐up in the femur (upper) and tibia (lower), grouped by experimental condition. Control groups: sham surgery and meniscectomy. Each combination of symbol and colour represents a different study. PCL, polycaprolactone (poly(ε‐caprolactone); PNAGA, poly(N‐acryloyl glycinamide); PNASC, poly(N‐acryloyl semicarbazide); PUA, polyurethane acrylate.

Chondroprotection was evaluated in all the selected studies except for one [33], using standardized scoring systems, including the International Cartilage Repair Society (ICRS) [11, 12, 35, 37, 58, 59, 69] (Figure 8), the Mankin score [11, 12, 17, 20, 35, 37, 39, 58, 59, 69] (Figure 9) or the Osteoarthritis Research Society International (OARSI) score [61, 64, 65, 66, 67, 68] (Figure 10).

Overall, the experimental groups implanted with functionalized scaffolds demonstrated reduced cartilage degeneration compared to unmodified constructs and meniscectomy controls, which showed the highest degeneration. An exception was a PNASC/PNAGA scaffold that achieved OARSI scores not significantly different from sham controls [68].

ICRS scoring revealed improved preservation of cartilage surfaces in scaffold‐treated joints, with performance varying according to biomaterial composition and functionalization (Figure 8). Unmodified PCL scaffolds showed moderate degeneration, including cartilage thinning and occasional subchondral bone exposure [6, 24]. BM‐MSC–seeded PCL scaffolds reduced cartilage damage compared with unseeded constructs, although chondroprotection remained lower than sham groups [12, 59, 69]. Similar results were obtained by protein‐based modifications and graphene‐functionalized scaffolds at 6 months [37, 58]. Non‐resorbable PUA‐based scaffolds also demonstrated minimal cartilage degeneration, with comparable outcomes to functionalized PCL constructs [11].

Mankin score analysis confirmed these findings, with the lowest scores observed in biofunctionalized scaffolds, including MECM‐containing constructs and BM‐MSC–seeded PCL scaffolds, which preserved cartilage architecture more effectively than unmodified PCL (Figure 9) [12, 17, 35, 37, 39, 58, 59, 69].

Although less frequently used, the OARSI scoring further supported the chondroprotective effect of engineered scaffolds. PCL‐hydrogel hybrids [67], PLDLA‐TMC [27] and PNASC/PNAGA [68] scaffolds exhibited significantly lower scores than meniscectomy controls, indicating cartilage preservation, with one study [68] reporting comparable scores to those of the sham groups.

### Risk of bias and quality of evidence

The risk of bias assessment was conducted using the SYRCLE Risk of Bias tool (Supporting Information S1: Table 3) [21]. Overall, the methodological quality of the included studies was heterogeneous and characterized by a high proportion of items rated as ‘unclear’, indicating incomplete reporting of key experimental procedures. In most studies, random sequence generation was not described, and allocation of animals to experimental groups was frequently not reported (87%). Only a minority of studies reported randomized housing or blinded outcome assessment. Blinding of caregivers and evaluators was largely absent or not clearly reported, one of the most significant methodological limitations. Baseline characteristics of the animals were generally balanced (predominantly classified as ‘Yes’), and incomplete outcome data were uncommon. Most studies reported expected outcomes, suggesting a relatively low risk of selective reporting. Other potential sources of bias, such as conflicts of interest or funding, were not identified, although these aspects were not consistently discussed. Overall, the limited reporting of randomization, allocation concealment and blinding indicates a non‐negligible risk of bias. These gaps affect the reproducibility and cross‐study comparability of preclinical findings and should be addressed to increase the translational reliability of 3D‐printed meniscal scaffolds.

## DISCUSSION

3D‐printed meniscal scaffolds represent emerging regenerative strategies developed to address the biomechanical and biological limitations of conventional meniscal repair strategies. Although technological progress in additive manufacturing and biomaterial engineering has expanded the design possibilities for meniscal substitutes, their translation into clinical practice remains limited, underscoring the need for optimized material design and functional performance [4, 8, 15, 57]. The present systematic review evaluated the available preclinical in vivo evidence focusing on the interplay between material composition, biofunctionalization strategies, additive manufacturing techniques, scaffold mechanics and biological outcomes. Overall, while most studies reported encouraging regenerative responses, a substantial heterogeneity in biomaterial design, experimental models and outcome reporting was observed, as highlighted by the risk of bias analysis. This limits the direct comparison between the different experimental approaches and precludes the identification of optimal design principles.

### Biomaterials for meniscal scaffolds

The predominant use of resorbable polymers reflects the priority given to the regeneration process in meniscal tissue engineering. To be effective, these approaches need to balance the degradation process to ensure mechanical support until the regenerated tissue is fully competent. Among these materials, PCL emerged as the most widely adopted polymer due to its low melting temperature, which facilitates additive manufacturing, as well as its established biocompatibility, biodegradability and mechanical stability. However, the intrinsic and well‐known bioinertness of PCL requires additional strategies to promote cell–material interactions and tissue integration [43]. Several studies therefore explored various functionalization strategies, including cell seeding, MECM incorporation, growth factor delivery or chemical modifications [11, 12, 17, 20, 33, 36, 37, 67]. These approaches generally enhanced the biological performance of the scaffolds compared with meniscectomy controls and were consistently associated with progressive increases in mechanical properties over time, likely reflecting cellular infiltration and ECM deposition within the scaffold architecture. Importantly, PCL‐based constructs generally demonstrated favourable biocompatibility, with only transient inflammatory responses during the early postoperative phase [37]. Similar positive outcomes were also shown with PLDLA‐TMC resorbable scaffolds, which also supported fibrocartilaginous tissue formation without the need for exogenous growth factors [27].

Beyond these resorbable systems, non‐resorbable polymers have been investigated to provide sustained mechanical support and more tailored functional properties. PUA, used as an elastomeric scaffold component or in combination with bioactive hydrogels, supported fibrocartilage regeneration, GAG deposition comparable to native tissue and reduced cartilage degeneration [61, 65]. Other polymer formulations have been designed to replicate the anisotropic architecture of the native meniscus through controlled fibre alignment and pore size and morphology. For instance, DA crosslinking in PCU‐DA scaffolds enhanced mechanical stability by preserving hard‐domain integrity under physiological conditions [66]. Altogether, these findings highlight how polymer chemistry and network architecture influence scaffold degradation behaviour, mechanical competence and host response. Rather than acting as passive templates, biomaterials in these constructs are bioactive platforms that can regulate tissue regeneration through their physicochemical and structural properties.

### Biofunctionalization strategies

Most synthetic degradable polymers lack intrinsic biological activity; hence, biofunctionalization strategies have been widely adopted.

Cell‐based approaches predominantly employed BM‐MSCs and meniscal fibrochondrocytes. When combined with advanced PCL architectures, such as gradient biomaterial patterns or microsphere‐mediated delivery systems, BM‐MSC–seeded constructs promoted region‐specific ECM organization and vascular ingrowth [12, 59]. Similarly, PCL seeded with fibrochondrocytes supported neotissue formation and progressive mechanical maturation, although long‐term restoration of native meniscal microstructure remained incomplete [6, 24].

Alternatively, cell‐free strategies aimed to stimulate endogenous regeneration through biomimetic cues. MECM‐functionalized scaffolds promoted host cell infiltration and supported the formation of tissue with biomechanical properties similar to native meniscus [6, 33]. The incorporation of bioactive molecules, including KGN and PDGF‐BB, further enhanced endogenous cell homing and chondrogenic differentiation, while multi‐factor delivery systems enabled spatially controlled induction of fibrochondrogenic phenotypes within a single construct [20, 31, 33]. Hybrid scaffolds combining structural PCL with hydrogels acted as carriers for bioactive molecules or ions, facilitating localized modulation of the regenerative microenvironment and promoting region‐specific tissue formation and reduced cartilage damage [34].

Despite these promising outcomes, the literature remains highly fragmented. Differences in functionalization strategies, experimental design and outcome measures precluded robust comparisons between cell‐based and cell‐free constructs. Furthermore, regulatory considerations associated with the clinical use of cellularized constructs may limit their translability [30, 54, 62].

### 3D‐Printing approaches

Additive manufacturing technologies play a pivotal role in defining scaffold architecture and, consequently, functional performance. FDM resulted in the most widely used, especially for PCL‐based scaffolds, due to its cost‐effectiveness and ability to produce complex geometries with controlled porosity and fibre orientation [6, 12, 24, 27, 31, 33, 34, 37, 39, 66, 67, 69]. Through careful tuning of printing parameters, FDM allowed the fabrication of anisotropic architectures that guided directional cell proliferation and partially reproduced the structural organization of the native meniscus. It also enabled the development of scaffolds with additional functionalities, such as the incorporation of DA crosslinks in PCU models, which improved elastomeric robustness under physiological conditions while preserving thermal adaptability during the processing [66]. Nevertheless, the range of printable bioactive materials compatible with this technique remains relatively limited.

Extrusion‐based bioprinting allowed the fabrication of hydrogel‐based and cell‐laden constructs under physiological conditions, allowing spatially controlled deposition of cells and bioactive molecules [35, 64]. When combined with structural polymer skeletons, this approach facilitated the generation of region‐specific biological signalling and collagen organization patterns resembling native meniscal tissue [20, 58, 59].

Complementary fabrication techniques have also been explored. Wet electrospinning generated nanofibrous PCL scaffolds with ultrastructural features unachievable through FDM [36]. However, control of fibre orientation with this technique is limited, leading to disorganized spatial distribution of cell proliferation [51, 52]. High‐resolution approaches such as DLP and DLW provided improved microarchitectural precision and mechanical resilience, allowing the fabrication of anisotropic and functionally optimized polymer networks [11, 68].

Overall, these findings demonstrate that manufacturing technology is a critical determinant of scaffold hierarchy and biological performance. The integration of high‐resolution fabrication techniques with advanced biomaterial design will likely be essential for generating reproducible and biomimetic meniscal constructs.

### Biomechanical properties of 3D‐printed meniscal scaffolds

Restoration of meniscal function requires not only anatomical replacement but also reproduction of its complex anisotropic mechanical behaviour. Achieving a balance between tensile resistance, compressive compliance and long‐term structural stability represents a major design challenge.

Although many studies aimed to approximate native mechanical properties, only one construct simultaneously achieved tensile and compressive moduli within reported physiological ranges [11]. However, these reference values themselves varied considerably in the literature (tensile modulus ranging from 54.3 to 120.1 MPa, compressive modulus 3.7–23.3 MPa), due to inconsistencies in testing and sampling protocols and the intrinsic regional heterogeneity of the native meniscus [55, 60].

PCL‐based scaffolds demonstrated high versatility, enabling the modulation of anisotropic mechanical behaviour through architectural design and biofunctionalization. Emerging elastomeric systems, including PUA‐, PNASC/PNAGA‐ and PLDLA‐TMC‐based scaffolds, showed potential to achieve higher mechanical properties, approaching native values [11, 27, 64]. Importantly, scaffold mechanics were strongly influenced by structural parameters, such as porosity, region‐specific organization, chemical modifications and hydrogel–polymer composites [6, 33, 35, 39].

Previous human studies consistently highlighted a premature scaffold resorption as a major issue, which often lead to not sustained mechanical properties and poor clinical outcomes [19]. Form our analysis of the literature, several studies reported a dynamic evolution of scaffold mechanical properties over time, characterized by an initial decrease followed by partial recovery, likely associated with tissue ingrowth and ECM deposition [6, 11, 69]. Whether this behaviour can be consistently achieved in clinical settings remains uncertain and may depend on degradation kinetics and the capacity of biofunctional strategies to sustain effective tissue remodelling. Overall, our analysis indicates that mechanical optimization alone is insufficient. Successful scaffold design must integrate structural architecture, controlled degradation kinetics and biological activation rather than focusing solely on initial mechanical strength.

Beyond the scaffold's intrinsic material and structural properties, the integration of the 3D‐printed construct within the joint environment represents another critical, yet comparatively underexplored, determinant of successful translation (see ‘Scaffold design and production process’ section).Across the included studies, fixation was most commonly performed by suturing the scaffold to the anterior and posterior horn insertions and to the joint capsule, while a subset of studies reinforced this construct with transtibial bone tunnels. Although no included study directly compared fixation techniques, suture fixation augmented with transtibial tunnels appears, in our view, to represent the more robust approach, as it more closely reproduces the bone‐to‐bone fixation principles already validated in clinical meniscal allograft transplantation and may better withstand the cyclic loading experienced during early postoperative weight‐bearing. This interpretation is based on indirect comparison across heterogeneous studies rather than controlled head‐to‐head data, and dedicated biomechanical studies directly comparing fixation strategies are needed to substantiate it.

### Animal model

Preclinical animal models are essential to assess scaffold integration and functional performance prior to clinical translation. However, species‐specific anatomical, biomechanical and biological differences can strongly influence the interpretability and translational relevance of the outcomes [10, 48, 50]. The ideal experimental model for meniscal substitution should have joint dimensions and geometry comparable to humans, similar loading and biomechanics, low intrinsic healing capacity and comparable biological and inflammatory responses.

Rabbits represented the most commonly used model, largely due to their cost‐effectiveness and relatively similar anatomy, despite their small knee size. Importantly, its biomechanical loading patterns and relatively high intrinsic healing capacity may overestimate scaffold efficacy [48, 50]. Sheep, in contrast, are considered a more translationally relevant model, with similar meniscal size, vascularization and mechanical loading, although gait‐related differences in joint biomechanics remain [7, 10, 50]. A further limitation of the ovine model is behavioural: as a prey species, sheep tend to mask overt signs of pain and discomfort, so gait‐based lameness scoring may underestimate the true impact of the implanted scaffold on joint function [44]. Dogs, used only in one study, exhibit anatomical and histological similarities to humans, although if they have a more pronounced backward slope. However, distinct biomechanics and strong tissue response may confound outcome interpretation, while ethical and regulatory constraints limit their use [3].

These interspecies differences highlight that preclinical outcomes must be interpreted within the specific biological and mechanical environment of each model and cannot be directly extrapolated to human clinical scenarios.

### Chondroprotective role

A crucial goal of meniscal scaffolds is the prevention of progressive articular cartilage degeneration. While early clinical studies of first‐generation meniscal scaffolds did not demonstrate clear chondroprotective superiority over meniscectomy in humans, the preclinical studies here reviewed consistently reported improved cartilage preservation in the experimental groups [51].

Several factors may explain this discrepancy. First, the human clinical studies involved first‐generation scaffolds, manufactured with materials and technologies that can now be considered outdated compared with those used in the preclinical studies [26, 48]. In fact, 3D‐printed scaffolds frequently incorporated advanced design features and biofunctionalization strategies that enhance their biological integration and outcomes. In addition, early scaffold resorption, without parallel matrix neodeposition, may have compromised sustained mechanical support in human studies. In contrast, many preclinical studies reported progressive recellularization and deposition of ECM over time. Nevertheless, it should be considered that species‐specific healing capacity, particularly in rabbits, may amplify apparent regenerative response. Finally, patient‐related factors, including age, baseline cartilage status, activity level and the specific meniscal damage, can influence the clinical effectiveness of meniscal scaffolds, modulating the joint's response. Future research should therefore explore if patient‐specific 3D‐printed scaffolds can improve outcomes compared to off‐the‐shelf implants, and whether specific patient subpopulations may respond differently to the same implant to the same implant.

Taken together, while preclinical findings suggest that modern bioactive and structurally optimized scaffolds may confer chondroprotective benefits, long‐term translational validation through standardized, well‐controlled studies remains essential.

### Current research limitations and translational clinical potential

The findings from the present systematic review highlight significant progress in the design of 3D‐printed meniscal scaffolds, with several approaches achieving mechanical and histological features approaching those of native tissue. Advances in additive manufacturing, architectural customization and bioactive material integration have addressed some limitations of earlier scaffold generations, particularly in terms of internal fibre organization and biological responsiveness.

Despite these substantial advances, the current body of evidence remains fragmented. Comparative studies between different scaffold designs are scarce, and no studies directly compared scaffolds fabricated from different biomaterials. Moreover, the inconsistent reporting of experimental parameters and methodological approaches further limits the possibility of robust synthesis of the available results. This fragmentation inevitably delays the identification of optimal design principles and makes the effective translation toward clinical settings more challenging. To overcome this gap, we propose herein a structured set of reporting items aimed at improving reproducibility and enabling a more objective comparison of scaffold performance from both mechanical and biological perspectives. Establishing standardized evaluation criteria will be critical to accelerate technological refinement and facilitate responsible clinical translation of next‐generation meniscal biomaterials towards clinical application (Table 4).

**Table 4:** Checklist for standardized reporting on development and testing of 3D‐printed meniscal scaffolds.

Experimental step	Essential item	Recommended item	Reporting guidance
*Step 1—3D Model preparation*	E1.1: Imaging source or design software used		Describe imaging method, segmentation process, CAD workflow
		R1.1: Internal structure design	Report porosity, fibre orientation, stiffness gradient
		R1.2: Printing file preparation	Report G‐code generation, slicing software (e.g., Cura, Simplify3D)
*Step 2—Material selection*	E2.1: Polymer		Report polymer type, supplier, sterilization method, experimental protocols
	E2.2: Bioactive modifications		Describe modification procedures (e.g., cell seeding, drug delivery systems, chemical functionalization)
*Step 3—3D printing process*	E3.1: Printing method and key parameters		Printer logs (temperature, nozzle diameter/pressure, layer height)
	E3.2: Post‐processing		Processing report (e.g., crosslinking, surface functionalization, sterilization)
*Step 4—Mechanical testing*	E4.1: Tensile and compressive properties		Report tensile/compressive and aggregate modulus, tensile/compressive strength, uniaxial tensile testing
		R4.1: Other mechanical properties	Report viscoelastic properties, shear modulus, resilience and energy dissipation, fatigue resistance
*Step 5—Animal implantation*	E5.1: Animal species, age, sex		Veterinary record
	E5.2: Type of defect, surgical and fixation approach		Describe surgical procedures in detail
	E5.3: Follow‐up period		Veterinary and surgical record
		R5.1: Additional animal characteristics	Report number of animals, weight
		R5.2: Housing condition	Veterinary record (analgesia, antibiotics, fixation method)
*Step 5a—Ethics and study design*	E5a.1: Ethical approval, 3Rs compliance, randomization, blinding strategy and inclusion/exclusion criteria		Report ethic statement and study protocol
		R5a.1: Power calculation,	Report power analysis, study protocol
*Step 6—In vivo functional assessment*		R6.1: Post‐implantation imaging (MRI/CT)	Describe imaging protocols
		R6.2: Gait analysis, joint kinematics, behavioural/pain assessment	Report motion capture, behavioural scoring
*Step 7—Histological evaluation*	E7.1: Histological staining. At least one ECM‐specific (Safranin‐O/Fast Green, Masson's trichrome)	R7.1: Histological staining. At least one additional ECM‐specific	Describe the staining method
		R7.2: Sample preparation	Report fixation, decalcification, freezing and embedding procedures
	E7.2: Histological evaluation of the implanted scaffold and articular tissues		Provide evaluation and possibly quantification of relevant parameters (such as scaffold degradation, inflammatory response, ECM composition and remodelling, vascularization)
	E7.3: Histological grading		Provide at least one histological score (e.g., ICRS, Mankin, OARSI)
*Step 8—Statistical Analysis*	E8.1: Statistical test(s) used and level of significance		Report statistical protocol and software (e.g., SPSS, R, GraphPad, etc.)

*Note*: The list provides a structured guide to ensure methodological consistency and comprehensive data collection across all experimental stages.

Abbreviations: 3D, three‐dimensional; CAD, computer‐aided design; CT, computed tomography; E, essential item; ECM, extracellular matrix; ICRS, International Cartilage Repair Society; MRI, magnetic resonance imaging; OARSI, Osteoarthritis Research Society International; R, recommended item.

## CONCLUSIONS

This systematic review highlights that the regenerative capabilities of 3D‐printed meniscal scaffolds are primarily driven by the complex interplay between biomaterials, architecture, degradation kinetics and biological activation rather than by polymer selection alone. Scaffold performance in preclinical models appears to follow a dynamic trajectory, with early reductions in mechanical properties frequently followed by recovery associated with cell recruitment and ECM deposition. These observations reinforce the importance of designing constructs that provide initial mechanical competence while actively supporting progressive tissue maturation. In this context, bioactivation strategies (cellularization, biochemical cues integration, hydrogel‐polymer composites) emerge as critical modulators of this process. However, their individual contributions remain difficult to delineate due to substantial heterogeneity. Therefore, we propose a practical checklist to harmonize data reporting, improving reproducibility and comparability for future 3D‐printed meniscal scaffolds.

Beyond meniscal application, the conceptual framework emerging from this review, which integrates structural hierarchy, controlled degradation and biologically responsive design, may inform the development of 3D‐printed bioactive scaffolds for other load‐bearing tissues. Progress toward effective clinical translation will rely on the adoption of standardized, mechanistically informed research strategies capable of linking material design to long‐term functional integration.

## AUTHOR CONTRIBUTIONS

**Andrea Ferrero**: Data curation; formal analysis; methodology; writing—original draft. **Giuseppe Anzillotti**: Data curation; writing—original draft. **Pietro Conte**: Data curation; writing—original draft. **Simonetta Resta**: Data curation; formal analysis; methodology; writing—original draft. **Simone Micalizzi**: Data curation; formal analysis; methodology; writing—original draft. **Cecilia Cappelli**: Data curation; formal analysis; methodology; writing—original draft. **Nada Mansour**: Data curation; formal analysis; methodology; writing—original draft. **Paolo Oliva**: Funding acquisition; supervision; writing—review and editing. **Elizaveta Kon**: Funding acquisition; supervision; writing—review and editing. **Daniele D'Arrigo**: Conceptualization; funding acquisition; supervision; writing—review and editing.

## FUNDING INFORMATION

European Union—Next Generation EU—PNRR M6C2, Project PNRR‐MAD‐2022‐12375978, CUP:E23C22000770006.

## CONFLICT OF INTEREST STATEMENT

The authors declare no conflicts of interest.

## ETHICS STATEMENT

The authors have nothing to report.

## Supporting information

Supporting File 1

## Data Availability

The authors have nothing to report.
