# Supplementary material for: Toward functional integration of 3D‐printed meniscal scaffolds: A materials‐driven systematic review of preclinical evidence and translational framework
Source: J Exp Orthop. 2026 Sep 7;13(3):e70898. doi: 10.1002/jeo2.70898 (PMC13549368; doi:10.1002/jeo2.70898)
Supplement: Supplementary file 1 [file JEO2-13-e70898-s001.docx]

**Supplementary Material**

**Table S1:** Values of the tensile and compressive moduli in the native knee.

| **Native Mechanical Properties** | **Tensile Modulus [MPa]** | **Compressive Modulus**  **(*Aggregate modulus) [MPa]** |
| --- | --- | --- |
| Li H et al. 2021 [29] | 45.58 | 3.76 |
| Li Z et al. 2020 [30] | 121.9 | 47 |
| Ding G et al. 2022 [31] | 80.5 | 15.4 |
| Sun Y et al. 2021 [32] | 114.5 | 0.135* |
| Chen M et al. 2019 [33] | 45 | 3.6 |
| Sweigart MA et al. 2005 [24] | 156 | 0.510* |

**Derivation of Interquartile Ranges (IQR) for Native Mechanical Properties**

Tensile Modulus IQR: 54.3-120.1 MPa

Compression Modulus IQR: 3.7-23.3 MPa

The interquartile ranges (IQR) reported for the tensile modulus and compressive/aggregate modulus were calculated using the numerical data extracted from the studies included in Table S3. For each mechanical parameter, all reported values were compiled and ordered from lowest to highest. The first quartile (Q1) and third quartile (Q3) were then determined following standard statistical definitions. The IQR was defined as the interval between Q1 and Q3. This approach allowed us to summarize the variability of the available literature data and provide a robust representation of the central distribution of native mechanical properties

**Table S2**: Extended table with values of mechanical properties

| **Study (first author, journal, and year)** | **Polymers** | **3D Printing technique** | **Mechanical properties**  **(Tensile)** | **Mechanical properties**  **(Compressive)** | **Mechanical properties**  **(Others)** |
| --- | --- | --- | --- | --- | --- |
| ***RESORBABLE SCAFFOLDS*** |  |  |  |  |  |
| ***PCL Based*** |  |  |  |  |  |
| *Bone Marrow Stem Cells* |  |  |  |  |  |
| Zhang ZZ et al.  2017  [34] | PCL | FDM | *Pre-implant:* **Tensile Modulus**:  Scaffold Cell seed MSC: 45MPa  Scaffold free: 25MPa  **Ultimate Tensile Strenght:**  Scaffold Cell seed MSC: 18MPa  Scaffold free: 18MPa  *Post-implant:* **Tensile Modulus**:  Scaffold Cell seed MSC t(3m)=25MPa; t(6m)=32MPa;  Scaffold free t(0)=25MPa; t(3m)=13MPa; t(6m)=11MPa;  **Ultimate Tensile Strenght:**  Scaffold Cell seed MSC  t(3m)=10MPa; t(6m)=10MPa;  Scaffold free  t(3m)=9MPa; t(6m)=7MPa;  ^15^m^4^N^-1^s^-1^ | *Pre-implant:*  **Compressive Aggregate Modulus:**  Scaffold Cell seed MSC: 35KPa  Scaffold free: 13Kpa  *Post-implant:*  **Compressive Aggregate Modulus:**  Scaffold Cell seed MSC  t(3m)=43KPa; t(6m)=51KPa;  Scaffold free  t(3m)=17KPa; t(6m)=15KPa; | *Pre-implant:*  **Permeability:**  Scaffold Cell seed MSC: 4,2*10^-15^m^4^N^-1^s^-1^;  Scaffold free:4,3*10^-15^m^4^N^-1^s^-1^  *Post-implant:*  **Permeability:**  Scaffold Cell seed MSC:  t(t(3m)=1,8*10^-15^m^4^N^-1^s^-1^; t(6m)=1,6*10^-15^m^4^N^-1^s^-1^;  Scaffold free; t(3m)=2*10^-15^m^4^N^-1^s^-1^; t(6m)=2,1*10^-15^m^4^N^-1^s^-1^ |
| Sun Y et al.  2020  [41] | PCL | OPUS system (Novaprint, China) | *In vitro 3 month:*  **Tensile Modulus:**  Scaffold + CTGF & TGF 118MPa  **-Radial Tensile:**  40MPa  -**Circumeferential Tensile:**  INNER:81MPa  OUTER:103MPa  **Ultimate Tensile Strenght:**  Scaffold + CTGF & TGF 22MPa |  |  |
| Sun Y et al.  2021  [32] | PCL | OPUS system (Novaprint, China) | *In vivo 6 months:*  **Tensile** **Modulus**:  Native: 114,5 MPa  Control: 78,5MPa  TCM: 122,7MPa  **Ultimate Tensile Strength:**  Native: 28,0 MPa  Control: 14,6 MPa  TCM: 24,1 MPa  **Tensile Modulus Radial:**  Native: 48,3MPa  Control: 30,5MPa  TCM: 40,7MPa  **Circumeferential Tensile:**  *outer*  Native: 117,2 MPa  Control: 51,7 MPa  TCM: 107,6 MPa  *inner*  Native: 95,0 MPa  Control: 43,9 MPa  TCM: 85,5 MPa | *In vivo 6 month:*  **Aggregate Modulus:**  Native: 135kPa  Control: 79.7kPa  TCM: 147.38kPa |  |
| Du M et al.  2024  [38] | PCL | FDM | *Pre-implant:* **Tensile Modulus:**  Scaffold USSP  X:24MPa  Y25MPa  Scaffold USDP  X:7MPa  Y:9MPa  Scaffold GSDP  X:2MPa  Y:30MPa | *Pre-implant:*  **Compressive Modulus:**  Scaffold USSP  34MPa  Scaffold USDP  17MPa  Scaffold GSDP  40MPa |  |
| *Fibrochondrocytes* |  |  |  |  |  |
| Chen M et al.  2019  [33] | PCL | FDM | *Pre-implant:* **Hybrid Scaffold:**  Tensile Modulus: 34,64 ±2,34 MPa  *Post-implant:*  **Tensile Modulus:**  Scaffold+Hydrogel+MFC  t(3m)=30MPa;  t(6m)=37MPa;  Scaffold+Hydrogel  t(3m)=18MPa;  t(6m)=29MPa;  Scaffold  t(3m)=14MPa;  t(6m)=24MPa; | *Pre-implant:* **Hybrid Scaffold:**  Compressive Modulus: 6,54±0,88MPa  *Post-implant:*  **Compressive Modulus:**  Scaffold+Hydrogel+MFC  t(3m)=1,8MPa;  t(6m)=2,3MPa;  Scaffold+Hydrogel  t(3m)=0,9MPa;  t(6m)=1,7MPa;  Scaffold  t(3m)=0,6MPa;  t(6m)=0,9MPa; |  |
| Jeong HJ et al.  2021  [37] | PCL | FDM | *Pre-implant:* **Tensile Modulus**  Control Group: 10.46 ±1.42MPa  Exp. Group: 4.91 ± 0.27 MPa  *Post-implant:*  **Tensile Modulus**  Control Group: 11.04 ± 0.08 MPa (1.5 m), 11.30 ± 0.13 MPa (3 m)  Exp. Group: 5.14 ± 0.61 MPa (1.5 m), 7.79 ± 1.16 MPa (3 m) |  |  |
| *Meniscal Extracellular Matrix* |  |  |  |  |  |
| Guo W et al.  2021  [44] | PCL | FDM | *Pre-implant:* **Tensile Modulus**  Large pore aligned scaffold: 31 ± 4.5 MPa  Large pore unaligned scaffold: 17.5 ± 1.3 MPa  Small pore aligned scaffold 46.4 ± 14.4 MPa  Small pore unaligned scaffold: 29.5 ± 2.9 MPa | *Pre-implant:*  **Compressive modulus**  Large pore PCL scaffold: 16 ± 1.6 MPa  Large pore PCL-MECM scaffold: 19.8 ± 1.7 MPa  Small pore PCL scaffold 29.8 ± 6 MPa  Small pore PCL-MECM scaffold: 37.5 ± 19.4 MPa |  |
| Li H et al.  2021  [29] | PCL | FDM | *Pre-implant:* **Tensile Modulus**  Native rabbit: 45.58 ± 1.30 MPa  PCL scaffold: 20.20 ± 1.54 MPa  PCL/MEMC: 27.15 ± 1.90 MPa  PCL/MEMC/KNG µS: 27.46 ± 1.33 MPa | *Pre-implant:* **Compression Modulus**  Native rabbit: 3.76 ± 0.14 MPa  PCL scaffold: 3.98 ± 0.34 MPa  PCL/MEMC: 4.14 ± 0.18 MPa  PCL/MEMC/KNG µS: 4.59 ± 0.18 MPa |  |
| *Growth Factors* |  |  |  |  |  |
| Lee CH et al.  2014  [45] | PCL | FDM | **Pull-out strength:**  – Scaffold: 54.5 N  – Native partial graft: 39.8 N  **Tensile modulus:** comparable to native meniscus | **Dynamic compression (central region):**  E* (complex modulus): similar to native meniscus  E′, E″: matched native intermediate zone | **Friction coefficient:** matched native values |
| Hao L et al.  2022  [39] | PCL | Dual Nozzle Bioprinting | *Pre-implant:* **Tensile Modulus**  PGHE: 24.2 ± 3.25 MPa  PCL: 42.9 ± 6.25 MPa | *Pre-implant:*  **Compression Modulus**  PGHE: 12.9 ± 3.64 MPa  PCL: 18.8 ± 3.65 MPa |  |
| *Chemical Functionalization* |  |  |  |  |  |
| Li Y et al.  2020  [42] | PCL | Wet-Electrospun | *Pre-implant:*  **Tensile Modulus:**  –P: 13MPa  –SP:12,5MPa  –SP-Sr: 20,6MPa | *Pre-implant:*  **Compressive Modulus:**  –P: 0.06 MPa  –SP:0.08 MPa  –SP-Sr: 0.11 MPa |  |
| Li Z et al.  2020  [30] | PCL | FDM | *Pre-implant:*  **Tensile modulus (MPa):**  – SF: 0.336 ± 0.142  – PCL: 11.067 ± 3.363  – PCL-SF: 13.402 ± 3.119  *Post-implant:* **Tensile Modulus (MPa):** PS (6/12w): 36,2/76.2  PS-L7(6/12w):59.6/97.3  Native(6/12w):121.9/120.7 | *Pre-implant:*  **Compressive modulus (MPa):**  – SF: 0.081 ± 0.028  – PCL: 5.689 ± 0.760  – PCL-SF: 6.582 ± 0.645  *Post-implant:*  **Compressive Modulus (MPa):**  PS(6/12w):17.6/21.2  PS-L7(6/12w):24.2/32.6  Native(6/12w):47.6/47.2 | *Pre-implant:*  **Friction force (Pa):**  – PCL in HA: 195.2 ± 24.6  – SF in HA: 4.4 ± 0.6 |
| Ma H et al.  2024  [46] | PCL | FDM | *Pre-implant:*  **Tensile modulus (MPa):**  – PCL: 29.35 ± 5.54  – PCL-Fib: 61.45 ± 11.75 | *Pre-implant:* **Compressive modulus (MPa):**  – PCL: 26.91 ± 3.86  – PCL-Fib: 42.25 ± 5.47 | *Pre-implant:* **Porosity (%):**  – PCL: 47.68 ± 1.59  – PCL-Fib: 42.93 ± 0.88 |
| Li J et al.  2024  [40] | PCL + graphene 1% | Extrusion | *Pre-implant:*  **Tensile modulus (MPa):** 0.62 ± 0.04  **Ultimate tensile load (N):** 3.12 ± 0.28  *Post-implant (6 month):*  **Tensile modulus (MPa):** 1.14 ± 0.05  **Ultimate tensile load (N):** 5.24 ± 0.31 | *Pre-implant:* **Aggregate modulus** **(MPa):** 0.18 ± 0.03  *Post-implant (6 month):*  **Aggregate modulus** **(MPa):**  0.35 ± 0.02 | *Pre-implant:*  **Permeability (mm⁴/N·s)**: 0.68 ± 0.03  *Post-implant (6 month):* **Permeability (mm⁴/N·s)**: 0.44 ± 0.02 |
| *Hydrogel* |  |  |  |  |  |
| Zhang Q et al.  2022  [36] | PCL | FDM | *Pre-implant:* **Circumferential tensile strength:**  -PCL+ PNAGA: 3.40 ± 0.19 MPa  **Circumferential Tensile modulus:**  -PCL+ PNAGA: 20.15 ± 1.37 MPa  **Radial tensile strength:**  -PCL+ PNAGA: 1.41 ± 0.08 MPa  **Radial Modulus modulus:**  -PCL+ PNAGA: 10.43 ± 1.54 MP | *Pre-implant:*  **Compressive modulus:** -PCL+ PNAGA:1.11 ± 0.14 MPa |  |
| Li H et al.  2025  [43] | PCL | FDM | *Pre-implant:* **Tensile Modulus:**  -PCL: 30,1 MPa  -PSM: 37,6 MPa | *Pre-implant:* **Compressive Modulus:**  -PCL : 4,9 MPa  -PSM:7,4 MPa |  |
| *Other* |  |  |  |  |  |
| Komatsu D et al.  2024  [35] | PLDLA-TMC | FDM | **Tensile Modulus:** *Pre-implant:* 83.64 (±20.79) MPa *Post-implant:* 72.46 (±28.53) MPa (1m); 45.45 (±21.13) MPa (1.5m), 10.86 (±2.01MPa) (3m) |  |  |
|  |  |  |  |  |  |
| ***NON RESORBABLE SCAFFOLDS*** |  |  |  |  |  |
| ***Polyurethane-based*** |  |  |  |  |  |
| Ding G et al.  2022  [31] | PU | DIW | *Pre-implant:* **Tensile Modulus PU Scaffold 3.5K-50:**  -PU/PDA/KNG: 80.3 MPa  *Post-Implant:* **Tensile Modulus:** PU(3/6m):70.0/77.0  PU/PDAKGN(3/6m): 72.5/85.0  Native (3/6m): 75.0/86.0 | *Pre-implant:*  **Compressive Mod PU Scaffold 3.5K-50:**  -PU/PDA/KNG: 16.5 MPa  *Post-Implant:* **Compressive Modulus:** PU(3/6m):11.5/13  PU/PDAKGN(3/6m):13/16.5  Native (3/6m):14/16.8 |  |
| Yan W et al.  2024  [51] | PUA | DIW | **Tensile modulus (MPa):**  Elastomer (PUA/HEA/nHAP) : 14.1  Hydrogel (WPUA/MAAm): 0.40  Scaffold: variable modulus  **Tensile strength (MPa):**  Elastomer (PUA/HEA/Nhap): 14.9  Hydrogel (WPUA/MAAm): 0.58 |  |  |
| Tian F et al.  2024  [47] | PUA | DIW | *Pre-implant:* **Tensile modulus (MPa):**  Cured elastomer (1:1:0.6 ratio of DOUPUA:TFA:HAP): 5.4 ± 0.2  **Tensile strength (MPa):**  Cured elastomer (1:1:0.6 ratio of DOUPUA:TFA:HAP): 10.1 ± 0.4  Hydrogel (WPUA:NIPAM): 0.14 | *Pre-implant:*  **Compressive strength at 30% strain:**  Hydrogel (WPUA:NIPAM): 3.3 KPa  **Compressive modulus:**  Hydrogel (WPUA:NIPAM): 10.6 KPa | *Pre-implant:*  **Friction coefficient:**  Pure hydrogel: 0.12 – 0.18  PC loaded hydrogels: 0.02 – 0.04 |
| ***PNASC/PNAGA-based*** |  |  |  |  |  |
| Xu Z et al.  2023  [48] | PNASC/PNAGA | Extrusion | *Pre-implant:*  **Tensile Modulus:**  Peripheral region 87.28 ± 6.06 MPa  **Tensile strength:**  peripheral region: 3.24 ± 0.21 MPa | *Pre-implant:*  **Compressive mod:**  interior region: 2,11 ± 0.28 MPa  peripheral region: 41,89 ± 4,04 MPa | *Pre-implant:*  **Suture strength:**  suture strength 4.57 ± 0.36 N |
| Zhang Q et al.  2024  [49] | PNASC/PNAGA | DLP | **Tensile strength:**  100% PNASC prestretched (stretched to 100% strain) parallel to fibers orientation: 13.35 MPa ± 0.59 MPa  100% PNASC Prestretched perpendicular direction: 5.01 ± 0.07 MPa  100% PNASC not prestretched: 3.83 ± 0.21 MPa  **Tensile Modulus**  PNASC 50% prestretched: 131.58 ± 6.49 MPa  PNASC 100% prestretched: 126.25 ± 4.33 MPa  Not prestreched: 83.84 ± 6.46 MPa |  | Mechanical properties remained stable even after being immersed in phosphate buffer saline (PBD) solution under 37° for 28 days. |
| ***Other*** |  |  |  |  |  |
| Yang X et al.  2025  [50] | PCU + DA bonds | FDM | *Pre-implant:*  **Tensile Modulus:**  pl- PCUDA: 37,0 MPa | *Pre-implant:* **Compressive Modulus:**  pl-PCUDA: 1.73 ± 0.22 MPa |  |

**Table S3**: Risk of bias assessment with SYRCLE’s assessment tool.

| **Ref.** | **Sequence generation** | **Baseline characteristics** | **Allocation concealment** | **Random housing** | **Blinding (caregivers)** | **Random outcome assessment** | **Blinding (outcome)** | **Incomplete outcome data** | **Selective outcome reporting** | **Other sources of bias** |
| --- | --- | --- | --- | --- | --- | --- | --- | --- | --- | --- |
| **Jeong HJ et al.**  **2021**  **[37]** | NO | YES | NO | NO | UNCLEAR | UNCLEAR | NO | NO | YES | NO |
| **Guo W et al.**  **2021**  **[44]** | UNCLEAR | YES | NO | NO | UNCLEAR | UNCLEAR | NO | NO | YES | NO |
| **Li J et al.**  **2024**  **[40]** | UNCLEAR | YES | UNCLEAR | NO | UNCLEAR | YES | UNCLEAR | NO | YES | NO |
| **Li Y et al.**  **2020**  **[42]** | NO | YES | UNCLEAR | NO | UNCLEAR | UNCLEAR | NO | NO | YES | NO |
| **Li H et al.**  **2021**  **[29]** | NO | YES | NO | NO | UNCLEAR | UNCLEAR | NO | NO | YES | NO |
| **Xu Z et al.**  **2023**  **[50]** | UNCLEAR | YES | NO | NO | UNCLEAR | UNCLEAR | NO | NO | YES | NO |
| **Zhang ZZ et al.**  **2017**  **[34]** | NO | YES | NO | NO | UNCLEAR | UNCLEAR | UNCLEAR | NO | YES | NO |
| **Zhang Q et al.**  **2024**  **[49]** | UNCLEAR | YES | NO | NO | UNCLEAR | UNCLEAR | NO | NO | YES | NO |
| **Chen M et al.**  **2019**  **[33]** | UNCLEAR | YES | UNCLEAR | NO | UNCLEAR | UNCLEAR | NO | NO | YES | NO |
| **Sun Y et al.**  **2020**  **[41]** | NO | YES | UNCLEAR | NO | UNCLEAR | YES | UNCLEAR | NO | YES | NO |
| **Li H et al.**  **2025**  **[43]** | UNCLEAR | YES | UNCLEAR | NO | UNCLEAR | UNCLEAR | NO | NO | YES | NO |
| **Yan W et al.**  **2024**  **[51]** | NO | YES | UNCLEAR | NO | UNCLEAR | YES | UNCLEAR | NO | YES | NO |
| **Lee CH et al.**  **2014**  **[45]** | UNCLEAR | YES | UNCLEAR | NO | UNCLEAR | YES | UNCLEAR | NO | YES | NO |
| **Du M et al.**  **2024**  **[38]** | NO | YES | UNCLEAR | NO | UNCLEAR | YES | UNCLEAR | NO | YES | NO |
| **Ma H et al.**  **2024**  **[46]** | NO | YES | UNCLEAR | NO | UNCLEAR | UNCLEAR | NO | NO | YES | NO |
| **Tian F et al.**  **2024**  **[47]** | NO | YES | UNCLEAR | NO | UNCLEAR | YES | UNCLEAR | NO | YES | NO |
| **Zhang Q et al.**  **2022**  **[36]** | UNCLEAR | YES | UNCLEAR | NO | UNCLEAR | YES | UNCLEAR | NO | YES | NO |
| **Sun Y et al.**  **2021**  **[32]** | UNCLEAR | YES | UNCLEAR | NO | UNCLEAR | YES | UNCLEAR | NO | YES | NO |
| **Hao L et al.**  **2021**  **[39]** | UNCLEAR | YES | UNCLEAR | NO | UNCLEAR | YES | UNCLEAR | NO | YES | NO |
| **Li Z et al.**  **2020**  **[30]** | UNCLEAR | YES | NO | NO | UNCLEAR | UNCLEAR | NO | NO | YES | NO |
| **Ding G et al.**  **2022**  **[31]** | NO | YES | UNCLEAR | NO | UNCLEAR | YES | UNCLEAR | NO | YES | NO |
| **Yang X et al.**  **2025**  **50** | UNCLEAR | YES | UNCLEAR | NO | UNCLEAR | YES | YES | NO | YES | NO |
| **Komatsu D et al.**  **2024**  **[35]** | NO | YES | NO | NO | UNCLEAR | UNCLEAR | NO | NO | YES | NO |

**Bibliography**
